# Acetyl-CoA Metabolism and Histone Acetylation in the Regulation of Aging and Lifespan

**DOI:** 10.3390/antiox10040572

**Published:** 2021-04-08

**Authors:** Patrick C. Bradshaw

**Affiliations:** Department of Biomedical Sciences, James H. Quillen College of Medicine, East Tennessee State University, Johnson City, TN 37614, USA; bradshawp@etsu.edu; Tel.: +1-423-439-4767

**Keywords:** acetyl-CoA, aging, autophagy, acetylation, histone deacetylase, calorie restriction

## Abstract

Acetyl-CoA is a metabolite at the crossroads of central metabolism and the substrate of histone acetyltransferases regulating gene expression. In many tissues fasting or lifespan extending calorie restriction (CR) decreases glucose-derived metabolic flux through ATP-citrate lyase (ACLY) to reduce cytoplasmic acetyl-CoA levels to decrease activity of the p300 histone acetyltransferase (HAT) stimulating pro-longevity autophagy. Because of this, compounds that decrease cytoplasmic acetyl-CoA have been described as CR mimetics. But few authors have highlighted the potential longevity promoting roles of nuclear acetyl-CoA. For example, increasing nuclear acetyl-CoA levels increases histone acetylation and administration of class I histone deacetylase (HDAC) inhibitors increases longevity through increased histone acetylation. Therefore, increased nuclear acetyl-CoA likely plays an important role in promoting longevity. Although cytoplasmic acetyl-CoA synthetase 2 (ACSS2) promotes aging by decreasing autophagy in some peripheral tissues, increased glial AMPK activity or neuronal differentiation can stimulate ACSS2 nuclear translocation and chromatin association. ACSS2 nuclear translocation can result in increased activity of CREB binding protein (CBP), p300/CBP-associated factor (PCAF), and other HATs to increase histone acetylation on the promoter of neuroprotective genes including transcription factor EB (TFEB) target genes resulting in increased lysosomal biogenesis and autophagy. Much of what is known regarding acetyl-CoA metabolism and aging has come from pioneering studies with yeast, fruit flies, and nematodes. These studies have identified evolutionary conserved roles for histone acetylation in promoting longevity. Future studies should focus on the role of nuclear acetyl-CoA and histone acetylation in the control of hypothalamic inflammation, an important driver of organismal aging.

## 1. Introduction

Therapies are urgently needed to slow the development of aging-related diseases. Since dysfunctional central metabolism plays an important role in the pathogenesis of aging-related disorders, metabolic therapies are becoming increasingly viable options for their treatment. Successful pathology-delaying metabolic therapies such as calorie restriction (CR) [1], ketogenic diet [2,3], and intermittent fasting [4] have shown efficacy in pre-clinical models. These therapies decrease systemic glucose levels and glycolytic metabolism leading to adipose tissue lipolysis and hepatic ketogenesis [5]. To maintain cellular energy production in peripheral tissues, these therapies increase the mitochondrial oxidation of non-glucose-derived energy substrates such as fatty acids, ketone bodies, and branched chain amino acids. These fuels are broken down in the mitochondrial matrix primarily into acetyl-CoA, which stimulates citric acid cycle flux for energy production. Importantly, the metabolism of these fuels bypasses the pyruvate dehydrogenase complex (PDC), which converts pyruvate, mostly derived from glucose, and NAD^+^ into acetyl-CoA and NADH. But PDC becomes increasingly inactivated by phosphorylation in aging and many aging-related disorders [6]. This may be the result of a desperate attempt by the cell to shunt pyruvate through lactate dehydrogenase and increase NAD^+^ levels to support glycolysis when mitochondrial electron transport chain (ETC) hydrolysis of NADH to NAD^+^ declines [7]. The reduced NAD^+^/NADH redox couple and PDC inactivation may be partly responsible for the decreased acetyl-CoA levels and decreased histone acetylation [8] that may occur in the aged brain [9]. As discussed in more detail later, PDC is a major provider of acetate [10] for nuclear acetyl-CoA synthesis used for histone acetylation in the hippocampus [11]. In some cells all subunits of the PDC can translocate from mitochondria to the nucleus in response to cellular stress to provide acetyl-CoA for histone acetylation [12].

## 2. Acetyl-CoA and CoA-SH Levels

The acylated or non-acylated forms of the acyl-CoA/CoA-SH redox couple are used as a coenzyme in over 200 reactions [13], roughly 4% of the enzymatic reactions in the cell. Therefore, genetic manipulations or pharmacological treatments that alter this redox couple will affect the steady state levels of hundreds of metabolites, greatly re-wiring cellular metabolism. Cytoplasmic acetyl-CoA levels have been reported to be roughly 7 µM in rodent brain, while mitochondrial acetyl-CoA levels were reported to be just slightly higher at 10 µM [14]. Acetyl-CoA levels are difficult to measure in tissues, especially in brain and liver. Over 50% of hepatic acetyl-CoA was shown to degrade at room temperature just 30 s following euthanasia in rat [5]. So, a method of estimating hepatic acetyl-CoA was developed using the tight correlation between the turnover of [^13^C_4_] ß-hydroxybutyrate and hepatic acetyl-CoA levels [5].

In cell lines when 10 mM glucose was present, acetyl-CoA was measured to be in the range of 6–13 µM but dropped to 2–3 µM when 1 mM glucose was present [15]. In rodent tissues total CoA (CoA-SH plus CoA thioester-containing compounds) in mitochondria was 2 to 5 mM, but was much lower in the cytoplasm, at concentrations usually between 40 and 150 µM [16]. Given that mitochondria make up on average 10–15% of the volume of the cell, there is frequently more total CoA in mitochondria than in the cytoplasm of tissues. However, in transformed cells in culture, due to the Warburg effect decreasing oxidative metabolism, cellular CoA-SH levels are significantly less than those found in rodent tissues and similar to acetyl-CoA levels, at concentrations of roughly 5 to 15 µM [10]. During growth with 10 mM glucose the acetyl-CoA: CoA-SH ratio was 1.5–2 and this ratio dropped below 1 when the cells were cultured with 1 mM glucose [15].

In mouse liver acetyl-CoA levels increased by roughly 60% from 3 months to 30 months of age, while CoA-SH levels only increased by roughly 20% during this time [17]. Unlike the ADP/ATP ratio that is fairly static in most tissues, the acetyl-CoA/CoA-SH ratio can vary widely during fasting and feeding cycles and under different dietary and disease conditions [18], making it an effective modulator of cellular signaling. As an example of how the acetyl-CoA/CoA-SH ratio changes, cells from premature aging mitochondrial DNA mutator mice showed a 10-fold decrease in CoA-SH, but only a 20% decrease in acetyl-CoA [19], once again reflecting the loss of CoA-SH in actively dividing cells when mitochondrial oxidative metabolism is disrupted. However, in a clinical trial where ten patients with mitochondrial disorders were supplemented with a multi-vitamin containing 25 mg/day of pantothenate (vitamin B5), a precursor of CoA-SH, no improvements were found in disease symptoms [20].

## 3. CR Appears to Increase Hepatic Acetyl-CoA without Decreasing Autophagy, While Long Term Fasting Appears to Decrease Hepatic Acetyl-CoA to Stimulate Autophagy

During fasting, once systemic glycogen stores are depleted and circulating glucose levels decrease, the citric acid cycle intermediate oxaloacetate is redirected toward gluconeogenesis in the liver. This results in increased levels of mitochondrial acetyl-CoA, which under normal replete conditions condenses with oxaloacetate to form citrate, but during fasting is used for ketogenesis [21]. The systemic increase in glucagon and decrease in insulin levels under these conditions facilitates adipose tissue lipolysis [22], which further contributes to the increase in hepatic fatty acid oxidation and acetyl-CoA levels. After a day or two of very low to no carbohydrate consumption, peripheral tissues increase their capacity to oxidize ketone bodies allowing the liver to decrease gluconeogenic output [23]. This increases the levels of hepatic mitochondrial oxaloacetate which reacts with and likely decreases the level of mitochondrial acetyl-CoA leading to a rise in mitochondrial matrix citrate. Citrate is transported by the mitochondrial citrate carrier (SLC25A1) [24] into the cytoplasm where ATP-citrate lyase (ACLY) catalyzes the reaction of citrate with CoA-SH to form acetyl-CoA and oxaloacetate. Acetyl-CoA can freely diffuse into the nucleus providing the substrate for histone acetyltransferases (HATs) such as GCN5, PCAF, CBP (CREB binding protein), p300, and TIP60/KAT5. But ACLY has been shown to be under tight transcriptional [25] and post-translational [26] control resulting in decreased activity during fasting, low energy, or low carbohydrate, high-fat diets keeping cytoplasmic acetyl-CoA levels and fatty acid synthesis at a minimum to conserve energy [27]. In humans once fasting is initiated it may take roughly a week for peripheral tissues to fully adjust to the lower glucose levels and maximize the ability to perform fatty acid beta-oxidation and ketolysis [28]. During this time the rate of hepatic acetyl-CoA utilization for ketogenesis slowly increases in parallel eventually raising plasma levels of the ketone body beta-hydroxybutyrate (BHB) over 5 mM [23].

In liver the global acetylation of cytoplasmic and mitochondrial proteins decreased with aging [29]. This contrasts with results from heart and skeletal muscle where global protein acetylation increased with aging, in part due to decreased NAD^+^ levels with aging decreasing sirtuin deacetylase activity [30]. In CR liver, one group reported increased acetyl-CoA levels and increased protein acetylation in nuclear, cytoplasmic, and mitochondrial fractions with histone H3 acetylation being unchanged [31]. Another group studying CR mice reported decreased hepatic acetyl-CoA levels, but increased global protein acetylation [32]. These different results for the acetyl-CoA measurements may depend on circadian differences in acetyl-CoA levels [31] and the length of time mice were present in the fasted state when they were euthanized. When CR mice are fed, they finish their food rapidly and enter a fasted state until their next feeding. Therefore, CR feeding protocols can have similarities with intermittent fasting protocols. So, some benefits of CR may be due to physiological changes resulting from fasting such as increased systemic ketone body levels and the increased oxidation of ketone bodies in mitochondria for energy [33]. Age may also play a role in the effects of CR on acetyl-CoA levels, since no change in hepatic acetyl-CoA levels was found in young mice on a CR diet, but a 40% increase in hepatic acetyl-CoA levels was found in aged mice on a CR diet [17].

While there is increased autophagy in mouse liver following fasting for longer than 24 h [34] due to increased fibroblast growth factor 21 (FGF21) levels, histone H3K27 demethylation by JMJD3/KDM6B, activation of peroxisomal proliferator-activated receptor-α (PPAR-α), and increased expression of transcription factor EB (TFEB) [35], there have been reports that CR does not increase hepatic autophagy, but only prevented the aging-related loss of autophagy in rats [36]. Others have shown that hepatic autophagy did not decline with aging in rat and life-long CR had no effect on this [37]. Together this data suggest that CR or fasting less than 24 h may slightly increase liver acetyl-CoA levels that do not substantially alter the rate of autophagy, but fasting for longer than 24 h activates ketogenesis and likely leads to decreased hepatic acetyl-CoA levels that stimulate autophagy. It is unknown how CR increases hepatic acetyl-CoA levels without inhibiting autophagy, but it could be due to increased mitochondrial acetyl-CoA levels without changes in the cytoplasmic acetyl-CoA levels that regulate autophagy.

## 4. ACLY Synthesizes the Majority of Nucleocytoplasmic Acetyl-CoA, While Acetyl-CoA Synthetase 2 (ACSS2) Plays a Smaller but Important Role

**Figure 1:**
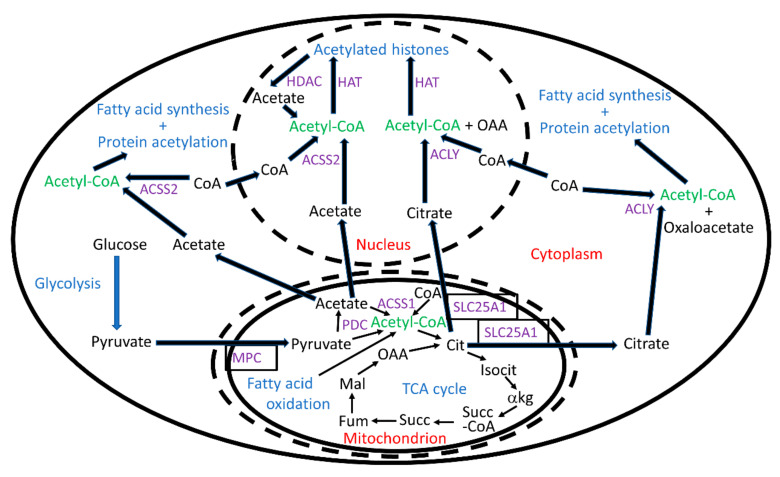
The substrates for the synthesis of cytoplasmic or nuclear acetyl-CoA by ACLY or ACSS2 are largely mitochondrial-synthesized citrate or acetate, respectively, although the acetate released from deacetylated nucleocytoplasmic proteins can also be an important substrate for ACSS2. Abbreviations: Cit, Citrate; Isocit, Isocitrate; α kg, Alpha-ketoglutarate; Succ-CoA. Succinyl-CoA; Succ, Succinate; Fum, Fumarate; Mal, Malate; OAA, Oxaloacetate; MPC, Mitochondrial pyruvate carrier; SLC25A1, Mitochondrial citrate carrier; PDC, Pyruvate dehydrogenase complex; ACLY, ATP-citrate lyase; ACSS1, Acetyl-CoA synthetase short-chain family member 1; ACSS2, Acetyl-CoA synthetase short-chain family member 2; and HAT, Histone acetyltransferase. Proteins are shown in purple font with membrane transporters boxed, while metabolites are shown in black font, except for acetyl-CoA in green font. Pathways are shown in blue font, while subcellular localizations are labeled in red.

ACLY is responsible for the vast majority of nucleocytoplasmic acetyl-CoA synthesis, while ACSS2, which uses acetate as the source for acetyl-CoA synthesis, plays only a minor role under normal conditions [38]. Therefore, not surprisingly, ACLY knockout mice are embryonic lethal [39], while ACSS2 knockout mice show only very mild phenotypes [40]. ACLY was also shown to be responsible for the vast majority of histone acetylation in murine embryonic fibroblasts (MEFs) [41]. In ACLY knockout MEFs adding a 1 mM acetate concentration to the cell culture medium stimulated cellular acetate uptake and ACSS2 activity to restore histone acetylation levels, but not the reduced rate of cellular proliferation. Brain ACLY expression in mice declined from embryogenesis through young adulthood reflecting the decreased need for lipid synthesis in postmitotic neurons and oligodendrocytes [39]. ACLY is expressed in both neurons and glia. In neurons it is localized almost exclusively to the cytoplasm [11], while in other cells it localizes to the nucleus and functions in DNA repair [42]. In adult brain ACLY expression was high in regions with high levels of cholinergic neurons, such as the hippocampus, brainstem, and spinal cord, where acetyl-CoA is used at high rates for acetylcholine synthesis [39]. The roles of ACLY and ACSS2 in cellular acetyl-CoA metabolism are shown in Figure 1.

**Figure 2:**
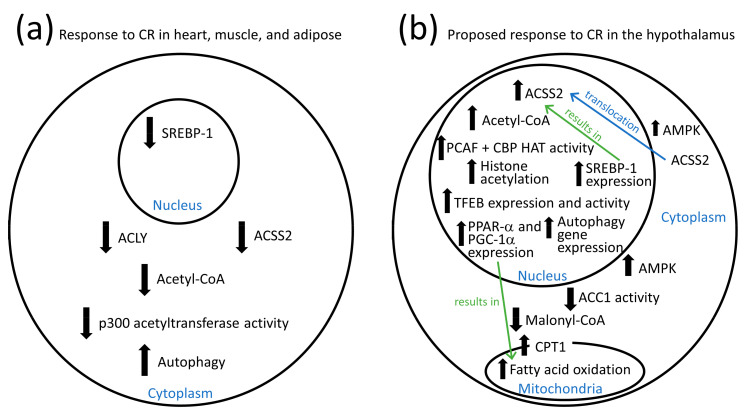
Acetyl-CoA may play different roles in the anti-aging effects of CR in different tissues. (**a**) CR results in decreased cytoplasmic acetyl-CoA levels in heart, skeletal muscle, and adipose tissue to stimulate autophagy to delay aging. (**b**) It is proposed that CR increases histone acetylation on specific promoters in some hypothalamic glial cells by activating AMP kinase (AMPK) that phosphorylates ACSS2 resulting in its nuclear translocation. ACSS2 associates with TFEB on promoters producing acetyl-CoA for PCAF and CBP-mediated histone acetylation and expression of autophagy genes. It is also proposed that the increased histone acetylation leads to increased PPAR-α expression and a shift to fatty acid oxidation. Abbreviations: SREBP-1, Sterol regulatory binding protein-1; ACLY, ATP-citrate lyase; ACSS2, acetyl-CoA synthetase short-chain family member 2; CBP, CREB binding protein; PCAF, p300/CBP-associated factor; HAT, Histone acetyltransferase; PPAR-α, Peroxisome proliferator-activated receptor-alpha; PGC-1α, PPAR gamma coactivator 1-alpha; ACC1, Acetyl-CoA carboxylase 1; and CPT1, Carnitine palmitoyltransferase 1.

ACLY gene expression is positively regulated by the SREBP-1 (sterol regulatory element binding protein-1) and ChREBP (carbohydrate response element binding protein) transcriptional regulators [43]. In several tissues such as liver and white adipose tissue, ACLY gene expression is downregulated when lipid synthesis is low such as during fasting, CR, or low carbohydrate, high-fat diets [44,45]. ACLY protein is stabilized by phosphorylation by several kinases such as AKT [46]. It is also stabilized by PCAF-mediated acetylation in response to high glucose [26], while SREBP-1 is destabilized by deacetylation by SIRT1 to decrease ACLY expression [47]. In tissues such as skeletal muscle, heart, and adipose tissue the fasting-induced decrease in ACLY expression and activity leads to decreased cytoplasmic acetyl-CoA levels, decreased p300 activity, and increased rates of autophagy [48] as shown in Figure 2a. High carbohydrate diets are often associated with negative health outcomes, while CR and ketogenic diets are frequently associated with healthy outcomes. Decreased ACLY activity may play a role in these positive effects [49]. Therefore, ACLY is a therapeutic target for many aging-related conditions including inflammatory diseases, neurodegenerative diseases, metabolic diseases, cardiovascular diseases, obesity, and cancer [50]. However, in mesenchymal stem cells ACLY appears to play a pro-longevity role as decreased expression of the mitochondrial citrate carrier SLC25A1 was shown to lead to decreased histone acetylation and stem cell aging. Adding acetate, the substrate for ACSS2, to the stem cell culture media was able to revert the aging phenotype [51].

There are times when ACSS2-mediated acetyl-CoA synthesis becomes very important. This was somewhat surprising given the relatively low plasma acetate levels in rodents and humans [52]. Serum acetate levels are normally 80–200 µM in non-fasted humans [52]. In rats serum acetate levels were shown to drop by 10% after one day of fasting and drop by 30% after three days of fasting [53], likely due to decreased acetate production by the intestinal microbiota [54], which in the fed state are the major source of serum acetate. Fasting increases the hepatic hydrolysis of acetyl-CoA to acetate by the enzyme acyl-CoA thioesterase 12 (ACOT12), formerly called acetyl-CoA hydrolase 1, leading to a two to three fold increase in the release of hepatic acetate into the bloodstream [52]. Therefore, acetate is released from liver in a similar way as ketone bodies during fasting. Serum acetate and ketone body levels are increased in diabetic patients [55,56], which may result in part from the increased hepatic CoA-SH [57] and acetyl-CoA levels in diabetic patients [58]. Serum acetate levels were found to decline slightly with aging [54].

Unexpectedly, it was discovered that intracellular levels of acetate are frequently high, on the same order of magnitude as pyruvate and lactate, at least when cells in culture were oxidizing glucose as the primary energy source. Elegant studies using isotope tracers identified the decarboxylation of pyruvate by PDC (see Figure 1) or alpha-ketoglutarate dehydrogenase as the major sources of cellular acetate with reactive oxygen species (ROS)-mediated pyruvate decarboxylation also playing a minor role [10]. Increased amounts of acetate were synthesized by PDC in the presence of pyruvate but in the absence of its other substrates CoA-SH or NAD^+^. Depletion of the PDC cofactor thiamine inhibited the synthesis of all PDC products including acetyl-CoA, acetate, and acetaldehyde [10]. Mitochondrial acetate and CoA-SH can be converted into acetyl-CoA by mitochondrial acetyl-CoA carboxylase 1 (ACSS1) for oxidation in the citric acid cycle [59]. Alternatively, acetic acid, the protonated form of acetate, can diffuse across the mitochondrial inner membrane [60] and into the nucleocytoplasmic compartment, where it dissociates back into acetate and can be used as a substrate for ACSS2 [10].

Some tumors, such as glioblastomas [61] and hepatocellular carcinomas [62] upregulate the expression of ACSS2 to capture acetate. Hepatocellular carcinoma cells cultured in normoxia synthesized acetyl-CoA that was shuttled to mitochondria for use as an essential energy source, while during hypoxia ACSS2 expression increased nearly five-fold [63], enhancing the rate of acetate utilization and the resultant acetyl-CoA synthesized was used to increase fatty acid synthesis and the acetylation of histone H3K9, H3K27, and H3K56 [64]. In non-small cell lung carcinoma cells ACSS2 showed increased Ser659 phosphorylation, a modification that stimulates ACSS2 nuclear translocation [65], when compared to the surrounding non-cancerous lung tissue [66]. ACSS2 knockout reduced tumor burden in mouse models of liver cancer [40]. However, decreased ACSS2 expression has also been associated with increased rates of metastasis and poor prognosis in some cancer patients [67]. Consumption of compounds that increase circulating acetate levels has been proposed as a therapy for glioblastoma [68,69], since acetate has been shown to arrest the division of glioblastoma-like stem cells [70].

Similar to the transcriptional regulation of ACLY, ACSS2 gene expression in liver, adipose, and intestine is controlled by ChREBP [43], while SREBPs also play an important role [71]. There are also Sp1 and Sp3 binding sites in the ACSS2 promoter [72]. ACSS2 expression in mouse liver and white adipose tissue decreased during fasting and increased during refeeding in a SREBP-1 dependent manner. Insulin also positively regulated ACSS2 expression, while streptozotocin treatment to induce type I diabetes and decrease insulin levels decreased ACSS2 expression [73]. Four weeks of a high-fat diet decreased ACSS2 and ACLY expression in liver and adipose, and decreased acetyl-CoA levels or the acetyl-CoA/CoA-SH ratio in both tissues. But the high-fat diet had no effect on hepatic histone acetylation levels [74]. Therefore, hepatic cellular acetyl-CoA levels can change independently of histone acetylation levels.

ACSS2 has been shown to contribute to fatty liver when mice consumed a high fructose diet. When animals quickly consumed large amounts of fructose, the fructose was not completely absorbed by the small intestine and the intestinal microbiota metabolized the fructose to acetate and other short chain fatty acids, which were released into the portal vein. This acetate was converted into acetyl-CoA by hepatic ACSS2 contributing to increased fatty acid synthesis and fatty liver [75]. Also, when fed a high-fat diet, ACSS2 knockout mice had less lipid deposition, gained less weight, and were protected from hepatic steatosis. However, the ACSS2 knockout mice were adversely affected by a 48-h fast. They lost more weight, had decreased locomotor activity, and were noticeably weaker. Following the fast, the ACSS2 knockout mice showed increased plasma levels of non-esterified fatty acids and decreased levels of glucose and ketone bodies suggesting a problem with hepatic fatty acid transport or beta-oxidation [40]. Because of these results, and others discussed below regarding ACSS2 function in brain, it is of interest to determine if ACSS2 knockout mice will be able to gain the health and longevity benefits conferred by CR.

## 5. ACSS2 Associates with Chromatin in Neural Cells and Provides Acetyl-CoA for Histone Acetylation That Induces Autophagy

Acetate metabolism in the brain was once thought to be almost strictly confined to astrocytes [76], as astrocytes provide roughly 10% of their energy needs by converting acetate into acetyl-CoA and then oxidizing the acetyl-CoA in the citric acid cycle [77,78]. More recent studies have shown moderate ACSS2 levels in neurons and oligodendrocytes, with the vast majority localized to the nucleus [79]. In a recent study the authors estimated that up to 30% of brain acetate metabolism can occur in neurons [80]. In mouse Cath.-a-differentiated (CAD) cells derived from catecholaminergic neural cells, ACSS2 translocated from the cytoplasm to the chromatin in the nucleus in response to serum deprivation and this was required for proper neuronal differentiation [11]. Following differentiation, ACSS2 co-immunoprecipitated with chromatin containing CBP and acetylated histone H3K9, H3K27, and H4K5 and acetylation was shown to be increased on histone H3K9, H3K27, H4K5, and H4K12, which was most prominent on the promoters of neuronal-specific genes. Interneurons deficient in CBP were also unable to successfully undergo neuronal differentiation [81,82], consistent with ACSS2 providing the acetyl-CoA for CBP during this process.

In hippocampal neurons due to the nuclear chromatin localization of ACSS2, acetate-derived acetyl-CoA leads to much greater histone acetylation than ACLY-derived acetyl-CoA [11]. However, this selective use of ACSS2-derived acetyl-CoA for histone acetylation does not occur in other cell types, such as cancer cell lines [15], due to a lack of nuclear or chromatin-localized ACSS2. For example, acetate administration to glioblastoma cells changed the expression level of 10% of the number of genes as supplementation with glucose (that results in citrate-derived acetyl-CoA), but of that 10% overlap in gene expression changes, there was a very strong concordance in the direction in which expression was changed [15]. This suggests that there was no major difference in the specificity of histone lysine residues acetylated between citrate or acetate-derived acetyl-CoA in these cells.

The ACSS2 gene is known to encode two transcripts that each has a unique transcriptional start site. One transcript called ACSS2-S1 encodes a protein with cytoplasmic localization and the other transcript ACSS2-S2 encodes a protein with a dual cytoplasmic and nuclear localization [83]. It will be important to determine if CR increases the relative abundance of ACSS2-S2 compared to ACSS-S1 or stimulates the association of ACSS2 with chromatin in the hypothalamus and hippocampus, which could potentially be responsible for some of the neuroprotective and anti-aging changes that result from CR and intermittent fasting [84]. An extensive two-part review of mammalian acetate metabolism and ACSS2 function has recently been published [52,85].

In glioma cells activation of AMPK by glucose restriction has been shown to stimulate the phosphorylation of cytoplasmic ACSS2 on Ser659 resulting in the translocation of ACSS2 to the nucleus. In the nucleus ACSS2 associates with TFEB, the major transcriptional regulator of autophagy and lysosomal biogenesis, and provides acetyl-CoA for increased histone H3K9 acetylation on the promoters of TFEB target genes to induce expression and increase the rate of autophagy [65]. TFEB is also known to induce the expression of peroxisome proliferator-activated receptor-alpha (PPAR-α) and peroxisome proliferator-activated receptor gamma coactivator 1-alpha (PGC-1α) [86], which stimulate mitochondrial fatty acid oxidation (Figure 2b) that is required for hypothalamic orexigenic signaling during fasting [87]. Consistent with this, a PPAR-α agonist was shown to increase food consumption in hypophagic mice deficient in hypothalamic fatty acid synthase [88]. PCAF is known to acetylate histone H3K9 and to stimulate autophagy [89,90,91,92] and therefore is a likely candidate to acetylate histone H3K9 on the TFEB promoter. Increased acetate levels have also been shown to stimulate the nuclear translocation of ACSS2 [93]. In addition, ACSS2 activity is inhibited by acetylation at lysine 661 and activated by deacetylation by SIRT1 [94]. Therefore, the ability of CR to increase NAD^+^ levels and activate sirtuins including SIRT1 [95,96] in the hypothalamus [97,98,99] may lead to acetyl-CoA synthesis for histone acetylation to stimulate autophagy and longevity.

Even though the nuclear pore allows free diffusion of acetyl-CoA between the cytoplasm and nucleus, nuclear synthesized acetyl-CoA often has preferential access to histones, while cytoplasmic acetyl-CoA frequently has preferential access to the enzymes of fatty acid synthesis. The spatial distribution of acetyl-CoA synthesizing enzymes and how it affects histone acetylation has been reviewed [100]. Activating AMPK to induce nuclear ACSS2 translocation [65], activating SIRT1 to increase ACSS2 activity, or increasing acetate levels through consumption of acetate precursors such as ethanol [101], N-acetyl-L-aspartate (NAA) [102], or glyceryl triacetate (GTA), also called triacetin, [70] could be used in the attempt to increase neuroprotective histone acetylation and gene expression in the brain. However, results with isotope-labeled ethanol or acetate administration in mice showed that this brain histones were only labeled by acetylation for roughly four hours [101]. So, longer lasting therapies are needed if this type of therapy is to be used for the treatment of aging-induced neurological disorders.

Different histone lysine residues can be acetylated in response to increased acetyl-CoA levels in different species or even in different tissues within the same species due to whether the Gcn5/PCAF family of HATs, the CBP/p300 family of HATs, or the MYST family of HATs has higher activity. Also, histone acetylation can occur to a greater extent in some cells by ACLY-derived acetyl-CoA and in some cells by ACSS2-derived acetyl-CoA. Consistent with this, the Gcn5/PCAF family, which acetylates histone H3K9, was shown to be highly stimulated by ACLY-derived acetyl-CoA, but less so by ACSS2-derived acetyl-CoA, in subcutaneous white adipose tissue or HCT116 colon cancer cells [41,103]. In slight contrast, the CBP/p300 family, which acetylates histone H3K27, and the GCN5/PCAF family were both stimulated by ACSS2-derived acetyl-CoA, but much less so by ACLY-derived acetyl-CoA in primary hippocampal neurons and differentiated CAD neurons [11].

Unlike PCAF, but like p300, the HAT GCN5 can function to inhibit autophagy. Overexpression of GCN5 in mammalian or *Drosophila* cells increased acetylation of the TFEB protein, which inactivated its function and led to impaired autophagy [104]. In a *Drosophila* tau model of neurodegeneration deletion of GCN5 prevented TFEB acetylation to stimulate autophagy and decrease pathology induced by tau overexpression [104]. Therefore, histone H3K9 acetylation on the promoters of TFEB target genes can increase their expression to increase autophagy, while direct acetylation of the TFEB protein by GCN5 inhibits its activity to decrease autophagy. The outcome may depend on the nuclear or cytoplasmic localization of ACSS2, TFEB, GCN5, and PCAF.

Knockdown of GCN5, but not knockdown of the p300 or MOF/KAT8 HATs stimulated apoptosis in neurons suggesting that the aging-induced loss of histone acetylation and increased susceptibility to neuronal apoptosis could be a result of decreased neuronal GCN5 activity [105]. The role of GCN5 in mammalian metabolism and energetics has recently been reviewed [106]. A negative feedback loop is present where stimulation of GCN5 activity by increased nucleocytoplasmic acetyl-CoA levels leads to acetylation of PGC-1α [107] and PGC-1ß [108] decreasing their activity to decrease mitochondrial biogenesis, which would help return acetyl-CoA levels back to normal by decreasing the production of the mitochondrial-synthesized acetate and citrate used as precursors for nucleocytoplasmic acetyl-CoA synthesis (see Figure 1).

## 6. Hypothalamic AMPK Increases with Fasting to Inhibit Fatty Acid Synthesis and Stimulate Fatty Acid Oxidation That Could Increase Nuclear Acetyl-CoA Levels to Stimulate HAT Function

The hypothalamus was shown to be a master regulator of the aging process. Decreasing hypothalamic inflammation by preventing NF-κB activation specifically in the hypothalamus led to a reduced rate of aging in mice, in part through preventing an aging-related decline in the release of gonadotropin releasing hormone (GnRH) [109]. Hypoxia-reoxygenation or the addition of tumor necrosis factor-alpha (TNF-α) to the hypothalamic GT1-7 neuronal cell line was shown to activate FOXO1 by preventing its phosphorylation to increase NF-κB activation to decrease GnRH secretion [110,111]. However, in mice lipopolysaccharide administration inhibited FOXO1 and activated hypothalamic NF-κB to induce inflammation and anorexia [112]. So, the role of FOXO1 in regulating NF-κB in the hypothalamus is still unresolved. However, hypothalamic FOXO1 levels were shown to increase in long-lived brain-specific IRS2-deficient mice that consumed more food and weighed more than the controls [113], suggesting that hypothalamic FOXO1 may contribute to longevity. SIRT1 overexpression in the brain leads to delayed aging due to its actions in the hypothalamus where it associates with Nkx2-1 to upregulate the expression of the orexin type 2 receptor (Ox2r) [97,98]. High caloric diets can lead to hypothalamic inflammation causing obesity [114,115]. Reduced hypothalamic inflammation is associated with lifespan extension in mice [116].

In most mammalian tissues and cell types activation of AMPK is protective, although there are exceptions [117], and AMPK activation may mediate some of the anti-aging properties of CR. But CR only appears to activate AMPK in a limited number of tissues including liver, skeletal muscle, and heart [118]. AMPK does not appear to be activated in the vast majority of the brain by anti-aging dietary interventions such as CR [118]. But, AMPK has been shown to be activated by fasting or high- fat diet in the hypothalamus [119,120,121,122], especially in the agouti-related protein (AgRP)-expressing neurons in the arcuate nucleus [123] and this stimulates food intake [124,125]. Therefore, given the positive association between CBP expression and longevity [126,127], it will be important to explore a potential role for the hypothalamic AMPK-ACSS2-CBP pathway in CR-induced longevity both in neurons and glia.

Malonyl-CoA, the product of acetyl-CoA carboxylase 1 (ACC1), a rate limiting enzyme in fatty acid synthesis, is an inhibitor of mitochondrial carnitine palmitoyl transferase 1 (CPT1), a rate limiting enzyme for fatty acid beta-oxidation. So, increased AMPK activity stimulates CPT1 to increase mitochondrial fatty acid β-oxidation. Hypothalamic neurons have high fatty acid beta-oxidation capacity [87], while neurons in other regions of the brain have limited fatty acid oxidation capacity [128,129]. Therefore, AMPK activation by fasting or CR is expected to maintain cytoplasmic acetyl-CoA levels in the hypothalamus, while cytoplasmic acetyl-CoA levels may drop in other brain regions. It is expected that during fasting AMPK-mediated nuclear translocation of ACSS2 in glial cells will increase nuclear acetyl-CoA levels in the hypothalamus. One group found that whole brain acetyl-CoA levels did not change following fasting [48], while another group found that brain acetyl-CoA levels declined slightly with fasting [130]. The decline in acetyl-CoA was shown to decrease p300 acetylation of the raptor subunit of the mTORC1 complex decreasing mTORC1 activity to stimulate autophagy [130,131]. Whole brain global protein acetylation levels did not change during CR [84], but CR may prevent the aging-related loss of AMPK activity [132] that likely contributes to organismal aging. ACLY is known to bind the alpha subunit of AMPK to inhibit its activity [133].

Mice subjected to 48 h of fasting showed decreased histone H3 acetylation in the ventromedial nucleus of the hypothalamus. These levels were not restored following seven days of refeeding and these mice showed an increased propensity to obesity when subsequently fed a high-fat diet [134]. Another study found that 16 h of fasting also decreased acetylation of histone H3K14 and H4K12 in the ventromedial nucleus of the hypothalamus, but not in POMC neurons of the arcuate nucleus [135]. There are many fiber projections between these two hypothalamic nuclei, which are responsible for their co-regulation of feeding and body weight [136]. The decreased histone acetylation results during fasting were somewhat unexpected given that fasting increases hypothalamic AMPK activity to decrease fatty acid synthesis and stimulates ACSS2 nuclear translocation in glial cells, which would likely prevent a decrease in nuclear acetyl-CoA levels. But fasting does not activate AMPK homogenously throughout the hypothalamus [137] and it is unknown how the expression and activity of ACLY, ACSS2, and many of the HATs and HDACs in the hypothalamus respond to fasting and CR. One study found that fasting in rats increased the hypothalamic expression of HDAC3 and HDAC4, while decreasing the expression of HDAC10 and HDAC11 [135]. More research is needed on fasting and CR-mediated histone acetylation changes on the promoters of specific longevity-regulating genes in the hypothalamus. It is likely that the acetylation of histone lysine residues on some promoters in the hypothalamus increases with CR because there was a fasting-induced increase in expression of the longevity gene FOXO3A [138]. Expression of FOXO3A is known to be induced by the increased levels of the ketone body and HDAC inhibitor BHB that occur during fasting [139].

## 7. Brain Histone Acetylation Changes with Aging

In the premature aging SAMP8 mouse model, brain acetyl-CoA levels and histone H3K9 acetylation levels declined with aging and pharmacologically activating AMPK led to the phosphorylation and inhibition of ACC1 to inhibit malonyl-CoA synthesis and fatty acid synthesis from malonyl-CoA. This led to a restoration of acetyl-CoA levels and nuclear histone H3K9 acetylation in neurons [9] and a roughly 30% increased lifespan [140]. Mechanisms that increase H3K9 acetylation and prevent histone H3K9 methylation in the hippocampus are also associated with enhanced cognitive performance in aged mice [141].

Histone lysine acetylation levels change with aging and comprehensive reviews have been published describing these changes and anti-aging dietary interventions [142,143], including those that occur specifically in aging brain [144]. In rodent brain, bulk histone H4 acetylation was shown to decrease with aging in Sprague-Dawley rat cortex [145] or Wistar rat hippocampus [146], but increase with aging in Long-Evans rat hippocampus [147]. Other researchers found that basal histone acetylation levels were not different in the hippocampus of mice between the ages of 3 months and 16 months. But there was an increase in histone acetylation following fear conditioning that occurred on histone H4K12 in 3-month, but not 16-month animals [148]. The 16-month old mice also showed a blunted behavioral response. Administration of the HDAC inhibitor butyrate or suberoylanilide hydroxamic acid (SAHA) were able to restore histone H4K12 levels and the magnitude of the behavioral response. HAT1 acetylates histone H4K12 and H4K5 in the cytoplasm before import into the nucleus [149,150]. HAT1 also plays an important pro-longevity role as HAT1 hemizygous mice show premature aging phenotypes such as mitochondrial dysfunction. HAT1 levels decline with aging in wild-type mice possibly facilitating the aging process [151].

Human and mouse brain prefrontal cortex genes that showed increased expression with aging were found to have broad histone H3K27 acetylation over the entire gene body and not just at the promoter in young adulthood. Many of these genes are involved in neuroinflammation. With aging the histone H3K27 acetylation marks along the gene body were lost leading to an upregulation of expression and increased inflammation [152]. Adding the HDAC inhibitor SAHA to 10-month mice for 3 months prevented the loss of histone H3K27 acetylation in the gene bodies and restored a more youthful gene expression pattern in the 13-month mice. These findings further highlight the importance of increasing the activity of brain CBP, a histone H3K27 HAT, and decreasing the activity of brain HDAC1 and HDAC2 starting in middle age to decrease neuroinflammation and promote healthy brain aging. CBP is also essential for memory consolidation in mice [153].

A study of histone post-translational modifications in postmortem young and aged human lateral temporal lobe of the brain identified decreased histone H4K20 acetylation in the aged brain [8]. The same study also examined Alzheimer’s disease (AD) brain and found increased histone H3K4, H3K9, and H3K27 acetylation compared to age-matched controls [8]. This contrasts with data obtained from several AD and other neurodegenerative disease animal models where decreased HAT activity has been measured [154]. Histone H4K16 acetylation was found to be increased with aging and decreased in AD when studying human lateral temporal lobe of the brain [155]. Another group studying human AD brain found decreased acetylation of histone H3K18 and H3K23 [156], while brain from a mouse model of AD showed decreased acetylation at histone H3K14, H4K5, and H4K12 [157]. The different results between studies may result from the different brain regions studied or the different methods and models used for histone acetylation measurements

## 8. CR Increases HAT and Decreases HDAC Activity in Brain Cortex

When isolated cortical neurons were cultured with low glucose to simulate fasting, supplementation with BHB increased CBP levels [158] and nuclear HAT activity and increased the association of the HAT p300 with NF-κB leading to increased expression of neuroprotective brain-derived neurotrophic factor (BDNF) [159]. However, p300 has also been shown to play roles in the induction of cellular senescence [160], which decreases organismal longevity [161]. CBP, which shares 63% amino acid sequence identity with p300 [162], can either stimulate [163] or inhibit [164] autophagy depending on the conditions. Consistent with p300 and CBP inducing an overlapping set of genes [165], overexpression of CBP in brain has also been shown to increase BDNF expression [166]. CR has been shown to increase HAT activity and decrease HDAC activity in brain cortex, in part, through increasing expression of miR-98-3p [84]. Increased expression of p300/CBP can lead to the inactivation of nuclear HDAC activity [167]. Consistent with this, CR was shown to decrease the striatal levels of HDAC1 in wild-type mice and HDAC2 in a Huntington’s disease mouse model [168]. Another group measured an increase in HDAC2 levels in the mouse hippocampus with aging that was prevented by CR [169]. In contrast to the hippocampal HDAC2 protein level measurements, whole brain HDAC2 mRNA levels (as well as the levels of HDAC3 and HDAC8) declined with murine aging, while HDAC1 mRNA levels did not change [170]. In mice from two months to eight months of age, histone acetylation was found to increase in white matter oligodendrocytes due a decrease in class I HDAC activity [171]. However, HDAC1 and HDAC2 protein levels were found to be increased in human brain white matter of elderly subjects [172].

## 9. CBP Levels in the Hypothalamus Positively Correlate with Longevity and This Is Likely Due to the Role of CBP in the Mitochondrial Unfolded Protein Response

CBP expression in the hypothalamus, but not hippocampus [173], has been shown to positively correlate with longevity across five mouse strains [127], while another group found that CBP expression in several tissues including hypothalamus, hippocampus, spleen, pituitary gland, and adrenal gland positively correlated with longevity [126]. Intermittent fasting was shown to increase the expression of CBP in mouse hypothalamus [168]. Consistent with this result, intermittent fasting has also been shown to increase the hypothalamic expression of the CBP target genes SREBP-1 and SREBP-2 [174,175] that could increase ACSS2 expression in hypothalamus to provide acetyl-CoA for CBP and other HATs. Fasting did not regulate SREBP-1 expression in cortex, while aging resulted in a 50% increase in SREBP-1 expression in both the hypothalamus and cortex [176].

Dietary interventions such as CR and intermittent fasting may activate CBP/p300 activity in brain by increasing the NAD^+^/NADH ratio, which would inactivate the NADH-dependent repressor CTBP1 that inhibits CBP/p300 function [177]. A discussion of possible mechanisms through which dietary restriction may increase CBP activity and histone acetylation in the hypothalamus to extend lifespan has been published [178]. Although p300/CBP activity did not decline in whole mouse brain with aging [179], rat cerebral cortex and hippocampal CBP protein levels were shown to decrease with aging [180]. The activity of p300/CBP decreased with aging in liver and skeletal muscle, perhaps as a mechanism to attempt to decrease the acetylation of the mTORC1 component raptor [130,131] and increase autophagy levels that decline with aging. The decreased CBP levels that occur with aging in some tissues may contribute to aging-induced pathology because haploinsufficiency of CBP due to hemizygosity results in human Rubinstein-Taybi syndrome, an autosomal dominant disorder characterized by many disease phenotypes including intellectual disability, shortness of stature, obesity, and heart and kidney defects [181]. CBP and p300 can also catalyze protein lysine beta-hydroxybutyrylation [182], while p300 was also shown to catalyze protein lysine propionylation, crotonylation, and butyrylation [183], although the catalytic activity decreases with increasing substrate carbon chain length [184]. So, acyl-CoA substrates besides acetyl-CoA may also contribute to the longevity effects associated with increased hypothalamic CBP levels. Exploring a role for CBP in catalyzing the beta-hydroxybutyrylation [185] of hypothalamic proteins, including histones, during fasting [186] and CR may be a fruitful area to identify novel mechanisms of cytoprotection.

Specific knockout of CBP in the hypothalamus resulted in obesity [187]. The obese phenotype of the hypothalamus-specific CBP knockout mice and Rubinstein-Taybi syndrome patients suggests an important role for CBP in satiety signaling [187]. The hypothalamic-specific CBP knockout mice showed decreased expression of BDNF and pro-opiomelanocortin (POMC), a polypeptide cleaved to release an α-melanocyte stimulating hormone (α-MSH) satiety signal, in the hypothalamus. Fasting also has an orexogenic effect and decreases hypothalamic POMC expression [188]. Hypothalamic global gene expression analysis of CBP deficiency showed a shift from glucose to fatty acid metabolism [187]. These phenotypes may be explained by activation of the FOXO1 transcriptional regulator when CBP activity was decreased. FOXO1 is inhibited from binding DNA by CBP-mediated acetylation [189] and sequestered in the cytoplasm by insulin receptor signaling/AKT-mediated phosphorylation [190,191]. FOXO1 induces the expression of pyruvate dehydrogenase kinase 4 (PDK4) [192,193]. PDK4 inhibits PDC activity to inhibit glycolytic metabolism, decrease malonyl-CoA levels, and induce fatty acid oxidation, which results in an orexigenic response. Consistent with this, overexpression of a constitutively active FOXO1 protein in the hypothalamus also led to hyperphagia and obesity [194]. FOXO1 was found to directly bind to the promoters to inhibit expression of POMC and activate expression of AgRP (agouti-related peptide), an orexigenic signal [195]. FOXO1 also increases expression of the orexigenic peptide neuropeptide Y [196].

FOXO1 is activated by post-translational covalent addition of O-linked N-acetylglucosamine (O-GlcNAc) [197,198]. In contrast to its overexpression, hypothalamic knockdown of FOXO1 [191] or AgRP neuron-specific knockout of the OGT (O-linked GlcNAc transferase) enzyme that adds O-GlcNAc to FOXO1 and other proteins induced an anorexigenic effect [199]. Therefore, the anti-aging ketogenic diet may induce an anorexigenic effect and promote the browning of white adipose tissue, which induces thermogenesis and weight loss, in part by decreasing plasma glucose levels to decrease AgRP neuron GlcNAc levels and FOXO1 O-GlcNAcylation and activity. Consistent with this hypothesis, AgRP neuron OGT activity has also been shown to prevent the browning of white adipose tissue during fasting to conserve energy [199]. The ketogenic diet promotes the browning of white adipose tissue [200,201], which could be due to decreased AgRP neuron GlcNAc levels and decreased O-GlcNAcylation of FOXO1. High-fat diets have been shown to decrease AgRP and orexin expression [202]. In contrast to high-fat diets, fasting stimulates hypothalamic AgRP neuron protein O-GlcNAcylation to stimulate an orexigenic response [199].

Overexpressing CPT1A, the rate limiting step in fatty acid oxidation, specifically in the hypothalamus also induced obesity mimicking the effect of CBP knockdown [187]. Consistent with the obese phenotypes of the hypothalamic CPT1A transgenic and hypothalamic CBP knockout mouse models, a shift to hypothalamic fatty acid metabolism was shown to be required for orexigenic signaling [87]. Hunger and satiety signaling are controlled by changes in hypothalamic malonyl-CoA levels. The increased malonyl-CoA levels that occur after feeding cause anorexigenic effects, while the decreased malonyl-CoA levels that occur during fasting cause orexigenic effects [203,204]. CPT1A overexpression likely induced the orexigenic response by decreasing malonyl-CoA levels. It is important to determine if decreased hypothalamic protein malonylation [205], including histone malonylation [206], plays any role in the anti-aging effects of CR.

Anorexigenic leptin receptor signaling decreases hypothalamic AMPK activity to increase ACC1 activity and malonyl-CoA synthesis from acetyl-CoA [207], while orexigenic ghrelin receptor signaling has the opposite effect to increase AMPK activity and decrease ACC1 activity and malonyl-CoA synthesis [137]. Ghrelin has shown efficacy for the treatment of several mouse models of neurodegenerative disease consistent with the neuroprotective effects of AMPK signaling [208,209]. Some of the beneficial effects of CR, such as the antitumorigenic effect [210], require neuropeptide Y synthesized in the hypothalamic arcuate nucleus. These effects may be mediated by ghrelin and neuropeptide Y stimulating hypothalamic autophagy [211,212], while the beneficial effects of CR on metabolism were shown to occur independently of ghrelin signaling [213]. The HDAC inhibitor butyrate was shown to increase CBP binding to the neuropeptide Y promoter and increase neuropeptide Y expression in the arcuate nucleus of the hypothalamus [214]. It is important to determine if increased hypothalamic nuclear ACSS2 and acetyl-CoA levels, CBP activity, and histone acetylation on TFEB target genes contribute to the beneficial effects mediated by CR.

**Figure 3:**
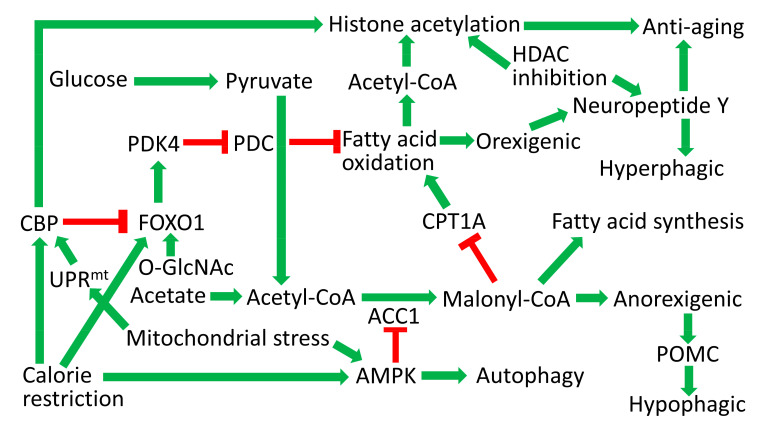
Hypothalamic acetyl-CoA, CBP, and histone acetylation play a central role in regulating organismal energy balance and longevity pathways. Abbreviations: CBP, CREB binding protein; O-GlcNAc, O-linked N-acetyl-glucosaminylation; FOXO1, forkhead box O 1; UPR^mt^, mitochondrial unfolded protein response; PDK4, pyruvate dehydrogenase kinase 4; PDC, pyruvate dehydrogenase complex; HDAC, histone deacetylase; ACC1, acetyl-CoA carboxylase 1; AMPK, adenosine monophosphate-activated protein kinase; POMC, pro-opiomelanocortin.

One mechanism through which CR likely delays aging is through modulation of hypothalamic mitochondrial function. In hypothalamic POMC neurons slight decreases in mitochondrial function, such as those that are induced by exercise, slight inhibition of mitochondrial protein synthesis, or knockout of apoptosis-inducing factor (AIF), stimulate mitohormesis and activation of the mitochondrial unfolded protein response (UPR^mt^). The activation of the POMC neuronal UPR^mt^ was associated with a shift from glucose metabolism to fatty acid metabolism and protection against obesity by stimulating UPR^mt^ and thermogenesis in white adipose tissue [215,216]. However, large decreases in POMC neuronal mitochondrial function inhibited hypothalamic fatty acid oxidation and caused obesity. CBP has recently been shown to be required for activation of the UPR^mt^ [126]. The protection against obesity in these paradigms of mitohormesis likely rely upon POMC neuronal UPR^mt^ activation and not upon the shift from glycolytic to fatty acid metabolism as the CBP and CPT1A mouse models described in the preceding paragraphs showed both obesity and the shift from glycolytic to fatty acid metabolism. Consistent with the UPR^mt^ being involved in longevity, hypothalamic levels of SATB1, homologous to the *C. elegans* DVE-1 UPR^mt^ transcriptional regulator, positively correlate with longevity in five mouse strains [127]. The central role of acetyl-CoA metabolism in hypothalamic energy balance and longevity pathways is shown in Figure 3.

One possible cause of hypothalamic aging is the increased expression of pyruvate dehydrogenase kinase 2 (PDK2) [217,218], the main PDK isoform present in brain [219], which results in a shift from glycolytic to fatty acid metabolism [220]. PDK4 is also expressed at a low level in the hypothalamus and its expression is increased by FOXO1 during fasting [138]. PDK2 and PDK4 are mainly expressed in astrocytes and microglia in the hypothalamus and PDK2 expression was increased by a high-fat diet through the transcriptional regulator PPARβ/PPARδ [219]. Activation of another PDK isoform, PDK1, in macrophages is known to stimulate the M1 pro-inflammatory phenotype [221] in part by decreasing the secretion of the anti-inflammatory cytokine interleukin-10 [222]. So, activation of PDK2 may stimulate the M1 response in hypothalamic microglia and a corresponding pro-inflammatory reactive A1 response in astrocytes as well. Consistent with this, ablating PDK2 in hypothalamic astrocytes was able to decrease hypothalamic inflammation and improve the disease phenotypes in a mouse model of diabetes [218]. PDK2 can also phosphorylate the mitochondrial rhomboid protease PARL to inhibit mitophagy [223] that could further contribute to its pro-inflammatory and pro-aging effects [224]. The inhibition of PDC by PDK2 may also lead to decreased cellular acetyl-CoA levels for histone acetylation affecting gene expression patterns.

The UPR^mt^ was identified and has been extensively studied in *C. elegans* [225]. The transcriptional regulator hypoxia-inducible factor-1 (HIF-1) was identified as being essential for UPR^mt^-induced longevity [226,227]. Increased HIF-1/HIF1α activity decreases mitochondrial ETC function [228,229], which may decrease the mitochondrial membrane potential to facilitate the induction of UPR^mt^. In mammals HIF1α was shown to be acetylated by 300/CBP [230] or PCAF [231,232] resulting in protein stabilization, while HDAC1 [230] and SIRT1 [231] were shown to remove the p300/CBP and PCAF-catalyzed acetylation marks, respectively. Similarly, CBP was shown to be required for the stabilization of HIF1α-containing multiprotein complexes that were competent to induce transcription [233]. Like CBP deficiency, HIF1α knockout in POMC neurons led to hyperphagia and obesity [234], likely due to decreased glycolytic activity and malonyl-CoA synthesis. CBP also acetylates HIF2α and stably associates with it depending on the acetyl-CoA produced by ACSS2 [235]. The acetylation of HIF2α is removed by SIRT1. All four proteins HIF2α, CBP, ACSS2, and SIRT1 were shown to be required for the induction of specific HIF2α transcriptional targets in response to low glucose levels. Therefore, this complex may play an important role in the hypothalamus to mediate the physiological effects of fasting and CR. Knockout of HIF2α specifically in hypothalamic POMC-producing neurons reduced the insulin-induced increase in POMC gene expression and led to aging-related hyperphagia, weight gain, and glucose intolerance [236]. Therefore, hypothalamic CBP, together with HIF1α, HIF2α, and ACSS2 may play an essential role in regulating central satiety signaling, inflammation, and aging.

## 10. Consumption of the Acetate Precursors GTA or Ethanol Increases Histone Acetylation in the Brain

There is evidence from humans that consuming acetate has an anorexogenic effect by stimulating POMC expression in the hypothalamus [237]. Consistent with this, increased acetate levels that occur in mice either by intraperitoneal acetate injection or by feeding the fermentable carbohydrate inulin led to decreased AMPK activity in the hypothalamus, increased malonyl-CoA levels, and decreased food consumption [238]. Hypothalamic expression of POMC was increased, while expression of AgRP was decreased 30 min after acetate injection. However, histone acetylation was not monitored. When acetate was intravenously-infused in mice only 3% of it localized to the brain, but the acetate preferentially accumulated in the hypothalamus [238]. Importantly, there was decreased phosphorylation of ACC1 by AMPK, which indicates increased ACC1 activity and malonyl-CoA synthesis and increased anorexigenic signaling.

Supplementation of rats with GTA was shown to double acetate levels in the brain, heart and liver [239]. GTA administration did not affect histone acetylation in liver, but the brain showed increased acetylation at histone H3K9, H4K8, and H4K16 [240,241]. Like CR, GTA administration led to increased brain HAT activity, decreased brain HDAC activity, decreased expression of HDAC2, and decreased neuroinflammation. However, another group found that GTA supplementation tripled plasma acetate levels, but brain acetate levels were not changed, although GTA administration increased expression of HDAC1 and HDAC2 in brain cortex [242]. In contrast to acetate-releasing compounds and hypothalamic CBP that stimulate anorexigenic signaling, CR stimulates orexigenic signaling. But all three of these therapies could potentially increase hypothalamic histone acetylation at specific promoters to stimulate anti-aging gene expression changes. More research is needed to determine hypothalamic changes in AMPK activity, acetyl-CoA levels, histone acetylation, and gene expression patterns resulting from GTA administration.

When NMDA receptor expression was pharmacologically decreased in mice, histone H3K9 and H4K8 levels declined. Increasing circulating acetate levels through either GTA supplementation or a probiotic, high fiber containing diet was shown to restore histone acetylation and increase the expression and activity of the brain NMDA receptor to restore mouse behavioral function [242,243]. Decreased NMDA receptor expression with aging was shown to be caused by decreased histone H3K27 acetylation levels due to decreased hippocampal cholesterol levels stimulating increased histone H3K27 methylation. Restoring cholesterol levels recruited CBP to the BDNF promoter restoring histone H3K27 acetylation and gene expression [244]. Since cholesterol is not permeable through the blood-brain barrier [245], therapies that inhibit brain cholesterol degradation may protect against aging-induced reduction of histone acetylation. Consistent with this data, different polymorphisms in APOE, a protein that transports cholesterol and other lipids in the brain and the bloodstream [245], are associated with extreme human longevity [246] and AD [247].

GTA administration was shown to be protective in experimental autoimmune encephalomyelitis, a mouse model of human multiple sclerosis [248]. GTA administration also improved rat motor function three days after traumatic brain injury [249]. GTA administration did not alter hippocampal mitochondrial biogenesis, but phosphocreatine levels were increased and AMP levels were decreased [250]. The absence of increased mitochondrial biogenesis was somewhat unexpected given that a ACSS2-CBP complex is highly enriched at promoters for the master transcriptional regulators of mitochondrial biogenesis NRF1 (nuclear respiratory factor 1) [251] and YY1 (Yin Yang 1) [11] in differentiated hippocampal neurons [11].

The label from injected ethanol was found on the transcriptional activating marks acetylated histone H3K9 and H3K27 in brain and to a similar extent in acetylated liver histones. However, acetylated skeletal muscle histones were labeled to a smaller extent, as ACSS2 is expressed at a lower level in mouse skeletal muscle compared to the other tissues examined. An acetate precursor such as GTA may need to be combined with a blood-brain barrier permeable HDAC inhibitor to help maintain neuroprotective brain histone acetylation levels. Administration of ketone ester, a CR mimetic, to mice has been shown to increase acetyl-CoA levels in brain [252], while a ketogenic diet has been shown to increase the mean lifespan of mice [2,3]. However, the success of therapies that increase acetyl-CoA levels in brain may be limited by increased acetyl-CoA levels and p300 activation in heart, liver, and white adipose tissue leading to decreased autophagy in these tissues and a pro-aging effect. Therefore, GTA and ketone ester could be administered during fasting or following a high-fat, low carbohydrate meal that decreases SREBP-1, ACSS2, and ACLY expression in heart, liver, and white adipose tissue and increases SREBP-1 expression and perhaps ACSS2 and ACLY expression in the hypothalamus. The glycerol in GTA may also lead to the synthesis of the toxic metabolite methylglyoxal, which has been associated with aging [253,254]. So, research on other potentially neuroprotective acetate-releasing compounds such as NAA should also be pursued. With this in mind, GTA administration was unable to restore decreased brain NAA levels [255]. It is not yet known if increasing brain acetate levels can mimic CR given that acetate stimulates anorexigenic signaling and CR stimulates orexigenic signaling. However, there is indirect evidence that some anorexigenic therapies could delay aging. For example, consumption of ketone ester has been shown to decrease ghrelin levels [256] that may induce anorexigenic signaling. In addition, knockout of the gene for the orexigenic peptide AgRP was shown to increase the lifespan of mice fed a high fat diet during late adulthood [257]. But more work is needed to confirm these preliminary findings and identify a molecular mechanism through which AgRP knockout extends lifespan.

## 11. Pyruvate, Ketone Bodies, Citric Acid Cycle Metabolites, or Pantothenate Supplementation May Increase Nucleocytoplasmic Acetyl-CoA Levels and Are Protective in Many Rodent Models of Human Aging-Related Disease

The administration of pyruvate or citric acid cycle metabolites potentially increases flux through the citric acid cycle to increase mitochondrial citrate efflux and ACLY-mediated nucleocytoplasmic acetyl-CoA synthesis for histone acetylation. Pyruvate can also be directly converted to acetate for ACSS2-mediated acetyl-CoA synthesis as mentioned earlier. Administration of pyruvate, the major precursor of both mitochondrial acetyl-CoA and cytoplasmic lactate, was shown to be protective in many disease models including epilepsy [258], ischemic injury [259], excitotoxicity [260], myocardial infarction [261], hemorrhagic shock [262], cardiogenic shock [263], and stroke [264]. Although it is likely that the increased synthesis of NAD^+^ by lactate dehydrogenase plays a protective role following pyruvate supplementation by stimulating central metabolism and sirtuin deacetylases, it should also be determined if increased acetate levels and histone acetylation play a protective role, especially for the treatment of neurodegenerative disorders.

Supplementation with other mitochondrial energy metabolites has also been shown to protect against epilepsy, including ketone bodies and the TCA cycle metabolites alpha-ketoglutarate (αkg) [265], succinate [266], and oxaloacetate [267]. Supplementation with TCA cycle metabolites results in anaplerosis and increased metabolic flexibility that stimulates cataplerosis including mitochondrial citrate efflux for nucleocytoplasmic acetyl-CoA synthesis. Oral administration of L-malate has been shown to improve memory in rats [268]. L-malate supplementation increased mitochondrial membrane potential, and the activities of the ETC complexes [269], similar to CR [270]. Pyruvate, αkg [271], isocitrate [272], and succinate [273] were all protective in Parkinson’s disease models. Pyruvate and αkg supplementation have also shown benefit in models of AD [274]. Increased levels of TCA cycle metabolites, like ketone bodies, increase energy production and bypass mitochondrial PDC to compensate for decreased glucose metabolism that occurs with aging and aging-induced disease [275]. This is especially important in most neurons that have limited fatty acid oxidation capacity [128,129]. It will be important to determine if increased nuclear acetyl-CoA levels and histone acetylation play a role in the neuroprotective effects of supplemented ketone bodies and TCA cycle intermediates.

Acetyl-CoA contains an acetyl group attached to a β-mercaptoethylamine group derived from cysteine, attached to a pantothenate group attached to an ADP group where a phosphate is present at its 3′-hydroxyl. In 1958 calcium pantothenate supplementation was shown to extend the lifespan of male C57BL/6 mice by 19% and decrease the weight loss that occurred following mid-life [276]. Pantothenate supplementation may increase lifespan through anti-inflammatory effects, as long-term pantothenate supplementation in humans was shown to decrease the plasma level of C-reactive protein (CRP), a marker of inflammation [277]. The anti-inflammatory effects could be due to a pantothenate-induced increase in the levels of protective microbiota such as *Bifidabacterium* in the intestine [278], which lead to the release of short chain fatty acids into the bloodstream. Pantothenate levels are higher in the plasma of elderly humans [279] and the livers of aged rats [280] suggesting an aging-induced impairment in cellular pantothenate uptake. Consistent with this, pantothenate levels were lower in blood cells from aged patients [281]. It is important to determine if the lifespan extension mediated by pantothenate supplementation is mediated by increased hypothalamic acetyl-CoA levels and histone acetylation. CoA-SH metabolism and its role in disease were recently reviewed [282].

## 12. Mammalian Changes in Histone Lysine Acetylation Marks with Aging outside the Brain

Aging-induced changes in histone acetylation have been well-studied in rats and mice [283]. For example, histone H3K14, H4K8, and H4K12 acetylation were increased during postovulatory aging of mouse oocytes [284]. In rat liver, histone H3K9 acetylation decreased slightly with aging [285], but some gene enhancers such as those for the gene CYP2E1, the major enzyme in brain that detoxifies ethanol to acetaldehyde [286], showed increased hepatic histone H3K9 acetylation with aging [287]. Measurements of total HAT activity in isolated rat liver nuclei showed a three-fold decrease with aging that was driven by the decline of the relatively strong activity of histone H4 acetylation in young rats, while the smaller hepatic histone H3 HAT activity increased by roughly 40% with aging [288].

Citrate and BHB levels were lower in serum from prematurely aging SAMP8 mice than aged-matched controls [289]. Decreased serum citrate may reflect decreased intracellular citrate and acetyl-CoA levels that may have led to the decreased brain histone H3K9 acetylation present in the aged SAMP8 mice [9]. The sirtuin SIRT6 deacetylates histone H3K9, which represses proinflammatory NF-κB signaling [290]. This modulation of inflammation may partly explain the premature aging phenotype of SIRT6 knockout mice [291] and the extended longevity of SIRT6 transgenic male mice [292]. So, histone H3K9 acetylation may be associated with aging or longevity depending on the specific genes and tissues affected. However, SIRT6 also deacetylates histone H3K18, H3K27, and H3K56 [293], which could also be involved in its pro-longevity effects [292,294]. For example, SIRT6 deacetylation of histone H3K18 has also been shown to prevent cellular senescence [294]. SIRT1 also deacetylates histone H3K9 [295] and both SIRT1 and SIRT2 can deacetylate histone H4K16 [58].

MEFs or bone marrow cells from short-lived Hutchinson-Gilford progeria model Zmpste24-deficient mice showed decreased histone H4K16 acetylation that was increased by treatment with the HDAC inhibitor butyrate [296]. Butyrate at 4 g/L, but not 8 g/L, in the drinking water increased mean lifespan from 22 to 26 weeks. The decreased histone H4K16 acetylation level in the MEFs was also reversed by overexpression of the MYST family HAT MOF/KAT8. Histone H4K16 acetylation in liver and kidney of wild type mice was also shown to decline with aging. The decreased abundance of histone H4K16 acetylation is associated with decreased expression of autophagy genes [297] and this could be partly responsible for the aging-related loss of autophagy that occurs in many tissues [298]. Therefore, therapies that increase MOF/KAT8 activity may help combat the phenotypes of aging. The ability of HDAC inhibitors to increase autophagy is likely mediated in part through increased histone H4K16 acetylation [299] that leads to FOXO1 gene expression. Increased FOXO1 activity results in sestrin 3-mediated mTOR inhibition [300] that leads to expression of autophagy genes [301]. The TIP60/KAT5 HAT can also acetylate histone H4K16 to stimulate FOXO1 expression and autophagy. In contrast to the autophagy-stimulating effects of histone H4K16 acetylation, increased acetylation of histone H3K56 results in autophagy inhibition [299]. Other mechanisms through which HDAC inhibitors stimulate autophagy include increased acetylation of ATG7, increased acetylation of p53, increased ROS production, and increased acetylation and activation of NF-κB [302]. Although CBP-mediated acetylation of lysine 310 of the RelA subunit of NF-κB may stimulate autophagy, it may play a pro-aging role by increasing pro-inflammatory gene expression. This acetylation event is opposed by SIRT1-mediated deacetylation [303].

In white adipose tissue from mice fed a high-fat diet, five histone acetylation marks positively correlated with acetyl-CoA levels including acetylated histone H3K9, H3K14, H3K23, H4K5, and H4K8, while histone H3K18 and H4K16 showed no correlation, and histone H4K12 showed a negative correlation with acetyl-CoA levels [74]. No significant effects on histone acetylation were seen after only five days on the high-fat diet. In cultured cells decreasing acetyl-CoA levels by ACLY knockdown did not alter acetylation of p53 by the HAT p300 even though histone acetylation was decreased [106,304]. Therefore, not all protein acetylation events are regulated uniformly by altered acetyl-CoA levels.

## 13. Yeast Changes in Histone Lysine Acetylation Marks with Aging and Anti-Aging CR

The yeast *Saccharomyces cerevisiae*, unlike metazoans, lacks the enzyme ACLY for synthesizing nucleocytoplasmic acetyl-CoA for histone acetylation [305]. Instead this yeast relies heavily upon the acetyl-CoA synthetase Acs2, homologous to ACSS2 in humans, for this function. Likewise, fungi lack homologs of the p300/CBP family of HATs, but contain a unique Rtt109 HAT, which lacks homologs in metazoans [306]. The Gcn5/PCAF family of HATs is conserved from yeast to mammals and is regulated by acetyl-CoA levels [108,307]. Total histone H3 and H4 protein levels declined with replicative aging in yeast although their mRNA levels increased. Replicative lifespan (RLS) was extended by histone H3 or H4 overexpression [308]. This data is consistent with the loss of transcriptional silencing with replicative aging in yeast [309].

In yeast, mutation of histone H3K14, a lysine that is normally acetylated, decreased RLS. Lifespan of the histone H3K14 mutant was further decreased by a mutation of histone H3K9, H3K18, or H3K23 [310]. Mutation of histone H3K9 by itself did not affect lifespan [311]. Adding glucose to yeast increased histone H3K9 and H3K14 acetylation through increased acetyl-CoA levels that stimulated the HAT Gcn5, part of the SAGA complex [312]. Others have shown that glucose addition induced acetylation of histone H3K9, H3K18, and H3K27 by the HAT Gcn5 and induced acetylation of histone H4K5, H4K8, and H4K12 by the HAT Esa1 [313]. Esa1 is homologous to mammalian TIP60/KAT5, a member of the MYST family of HATs and the NuA4 chromatin-modifying protein complex [314].

GCN5 deletion in yeast decreased chronological lifespan (CLS) when the cells were grown under standard laboratory carbon limited synthetic complete media conditions [315,316,317], but increased CLS when the cells were grown under winemaking conditions where nitrogen is limiting [318,319]. Under standard laboratory conditions GCN5 haploinsufficiency in diploid yeast or CR in a normal laboratory haploid strain extended RLS associated with moderately decreased histone H3K9 and H3K18 acetylation [320]. Overall, the data suggest that small to moderate decreases in histone H3K9 and H3K18 acetylation levels stimulate RLS or CLS, whereas larger decreases reduce longevity. It is possible that under the nitrogen limiting winemaking conditions the GCN5 deletion strain shows increased activity of another histone H3K9 or H3K18 HAT to increase histone acetylation back into the range just slightly below normal where CLS is increased. In this regard the HAT Rtt109 is known to acetylate not only histone H3K56, but also histone H3K9 [321].

The anti-aging compound spermidine was shown to inhibit HATs, likely Gcn5, to moderately decrease acetylation at histone H3K9, H3K14, and H3K18 to extend CLS [315]. The plant flavonoid epigallocatechin gallate (EGCG) also appears to extend CLS through partially inhibiting Gcn5 [320]. In contrast, Gcn5 has been shown to play a pro-longevity role in one strain of petite rho^0^ (mitochondrial DNA-deficient) yeast, as Gcn5 was required for the RLS extension induced by the retrograde response in this strain [322,323]. Experiments identified that Gcn5 and Sir2 were required to induce expression of the PHO84 plasma membrane high affinity phosphate transporter for lifespan extension to occur [322,324]. The mechanism through which Pho84 increases RLS is unclear, but its function has been linked with several longevity pathways including Tor/Sch9 [325,326], protein kinase A (PKA) [327,328], and the ER stress response [329,330]. Increased Pho84 function could also increase cellular phosphate levels that would inhibit the yeast mitochondrial permeability transition pore [331] to stabilize mitochondrial function.

In yeast inhibiting mitochondrial acetyl-CoA production led to a compensatory increased production of nucleocytoplasmic acetyl-CoA through upregulation of the expression of ACS2. The increased nuclear acetyl-CoA levels led to increased acetylation of histone lysine residues H3K9, H3K14, and H3K18 as well as hyperacetylation of histones H2A and H2B. However, histone H4K16 acetylation was unaltered. The increase in nucleocytoplasmic acetyl-CoA levels and histone acetylation resulted in decreased expression of autophagy genes and decreased CLS [332]. But somewhat paradoxically, acetyl-CoA levels decline during chronological aging in yeast and acetyl-CoA levels increase during lifespan extending CR [333]. The increase in acetyl-CoA levels during CR is mediated by increased expression of ACS2 and glucose-repressible ACS1 [334,335]. Since histone H3K9 and H3K18 acetylation levels decline and acetyl-CoA levels increase during CR [320], the decreased histone acetylation must be caused by either decreased HAT activity or by increased HDAC activity and not by the changes in acetyl-CoA levels that would tend to oppose these acetylation changes. In this regard, Sir2, which is activated by CR, has been shown to deacetylate histone H3K9 [336], while histone H3K18 can be deacetylated by the sirtuin Hst4 [337], the class I HDAC Rpd3 [337], and the class II HDAC Hda1 [338], although Hst4 only deacetylates histone H3K18 (as well as histone H3K23 and H4K12) under conditions of TORC1 inhibition [337], which occurs during CR [339].

Decreased levels of histone H4 amino-terminal acetylation have also been shown to partly mediate the increased RLS induced by CR in yeast [340]. Deletion of the Nat4 HAT, which acetylates the alpha-amino group on the first amino acid of histone H4, extended lifespan that was not further extended by CR. Histone H3K56 acetylation also decreases with yeast replicative aging [311]. Either increased or decreased histone H3K56 acetylation decreased RLS [308]. There is also increased acetylation of histone H4K16 at subtelomeric regions with replicative aging due to the decreased abundance of the Sir2 protein deacetylase with age. This increased histone H4K16 acetylation contributes to the loss of transcriptional silencing with aging [311]. Overexpressing Sir2 decreased histone H4K16 acetylation and increased RLS, while deletion of Sir2 had the opposite effects. Sas2 is the HAT that opposes Sir2 function on histone H4K16 [311].

Deletion of the Rpd3 HDAC had the opposite effect as deletion of Sir2 and increased RLS [341,342]. Rpd3 and Sir2 were later found to play antagonistic roles in transcriptional silencing [343]. Rpd3 can deacetylate several lysine residues in histones including histone H3K9, H3K18, H4K5 [344], H4K8, H4K12, and perhaps H4K16 [345], while another group showed that Rpd3 did not deacetylate histone H4K16 [346]. Histone H4K5, H4K8, and H4K12 are the residues most robustly deacetylated [344,347,348]. RPD3 mutants show a similar global gene expression profile as glucose limitation/CR [349], suggesting that increased acetylation of specific histone lysine residues, such as histone H4K5 and H4K12 [344,347,348], may partly be involved in mediating the longevity effects of RPD3 deletion and CR. Consistent with this hypothesis, Rpd3 is also a major target of the longevity promoting histone deacetylase (HDAC) inhibitor trichostatin A [349,350].

In yeast there are also strong links between histone acetylation, vacuolar pH, and RLS, as vacuolar pH increases with aging and CR delays this increase to extend lifespan [351]. Specifically, aged vacuoles with a higher pH take up less cysteine resulting in cytoplasmic toxicity, impaired cellular redox status, and decreased mitochondrial iron levels [352]. Somewhat counterintuitively, deletion of the HDAC RPD3, which extends RLS [342], stimulates the increased expression of NHX1, a vacuole sodium-proton exchanger that functions to increase vacuolar pH [349]. This result may be an example of hormesis, with small increases in vacuolar pH in young yeast cells, caused by increased NHX1 expression, resulting in a stress response leading to the increased ability to detoxify cytoplasmic cysteine and successfully prevent any further vacuolar pH changes with aging. Consistent with this interpretation, the ionophores nigericin, which mediates potassium-proton exchange, or monensin, which mediates sodium-proton exchange, slightly perturbed vacuolar function resulting in increased CLS in fission yeast, while vacuole proton pump deletion mutants showed decreased CLS [353], presumably due to larger non-hormetic effects on vacuolar pH and function.

A genome-wide screen of yeast viable single gene deletion strains was performed looking for strains with decreased histone H3K18 acetylation. Analysis of the hits from the screen identified the gene ontology category of vacuolar acidification as the top score with 8 different hits out of the 63 total deletion strain hits [354]. Therefore, changes in vacuolar/lysosomal pH may affect aging and lifespan through changes in histone acetylation. The authors suggested that alkalization of the vacuole results in acidification of the nucleocytoplasmic compartment that decreases HAT activity for histone H3K18. The enzymatic mechanism used by HATs includes a deprotonation step of an active site histidine that gives rise to pH-dependent changes in enzyme activity supporting such a hypothesis [355]. In contrast to the effects on HAT activity, nucleocytoplasmic acidification stimulates HDAC activity [356] that would further decrease levels of acetylation on the histones.

In contrast to yeast, increased cytoplasmic acetyl-CoA predominately stimulates acetylation of histone H3K27 in the plant *Arabidopsis thaliana* [357]. This is likely due in part to the fact that *Arabidopsis* contains several CBP/p300 HAT homologs absent in yeast [358] and that CBP/p300 HATs show preference for acetylating histone H3K18, H3K27, and H3K56 [359]. A heterodimeric ATP-citrate lyase enzyme complex is also present in *Arabidopsis* to synthesize nucleocytoplasmic acetyl-CoA [360].

## 14. Fruit Fly Changes in Histone Lysine Acetylation Marks with Aging and Anti-Aging Dietary Restriction

In 1948 supplementation of *Drosophila* with the CoA-SH precursor pantothenate was shown to increase lifespan [361]. More recently, *Drosophila* hemizygous for the HDAC Rpd3 were found to be long-lived and lifespan was not further extended by CR [362], suggesting an overlapping mechanism. Feeding the HDAC inhibitors butyrate or trichostatin A to *Drosophila* caused increased acetylation of histone H3 and lifespan, but no change was observed in histone H4 acetylation [363]. This is likely due to the much lower and less site-specific histone H4 HAT activity present in *Drosophila* compared to mammalian cells [364]. However, trichostatin A was found to increase histone H4 acetylation in *Drosophila* spermatids [365]. The result with fruit flies contrasts with the ability of butyrate or trichostatin A treatment to increase both histone H3 and H4 acetylation in mammalian cells [366,367]. In mammalian cells trichostatin A was shown to increase the acetylation of many acetylation marks including histone H3K9, H3K27 [368], H3K56 [369], H4K12 [370], H4K5 [371], H4K8, H4K16 [372] and H4K20 [373], although results are cell-type specific.

Knockdown of the *Drosophila* acetyl-CoA synthetase gene AcCoAS in brain resulted in increased autophagy levels and increased mean lifespan [332]. Therefore, brain AcCoAS appears to have an autophagy-inhibiting pro-aging role in *Drosophila* under standard conditions. So, the data from *Drosophila* does not completely align with data obtained from the use of a mammalian glia-derived cell line where AMPK was shown to activate ACSS2 to stimulate autophagy [65]. It will be important to determine if AMPK activation in brain can lead to the nuclear translocation of AcCoAS to induce autophagy and extend lifespan. Since only one acetyl-CoA synthetase gene is encoded by the *Drosophila* genome, unlike mammals that have two, it is possible that the vertebrate ACSS2 protein may have evolved chromatin-binding neuroprotective properties that are absent from *Drosophila* AcCoAS. But further research is needed to test this hypothesis. Both species also have ACSS3 homologs that show a mitochondrial localization like mammalian ACSS1, but preferentially bind propionate over acetate, to synthesize propionyl-CoA [374]. Further studies could determine if neuronal or glial-specific AcCoAS knockdown extends lifespan.

Uniquely, isolated *Drosophila* heads were shown to have an increased oxygen consumption rate (OCR) and acetyl-CoA levels in mid-life [375]. In mid-life flies, histone acetylation was shown to increase at histone H3K9, H3K9/14, H3K23, and H4K12 whereas histone acetylation was reduced at histone H3K18 and H4K8. Flies hemizygous for the ATP-citrate lyase gene ATPCL showed a 20% reduction in ATPCL activity, had reduced acetyl-CoA levels, and histone acetylation was blunted slightly on histone H3K14, H3K9/14, H3K23, and H4K12. The ATPCL hemizygous flies also showed a 32% increase in median survival. Flies hemizygous for the histone MYST family H4K12 acetyltransferase Chameau showed decreased acetylation specifically at histone H4K12 and a 24% increase in median lifespan. Double hemizygous ATPCL/Chameau mutant flies did not show a further extension of lifespan compared to the ATPCL hemizygous flies suggesting that the two interventions extend lifespan by a similar mechanism. Administration of the HDAC inhibitors butyrate or trichostatin A also increased the OCR of isolated *Drosophila* heads [375,376] showing that histone acetylation increases mitochondrial oxidative metabolism in brain.

Much research supports a neuroprotective role for *Drosophila* CBP (dCBP)/nejire. For example, CBP was protective in a *Drosophila* eye model of Alzheimer’s amyloid-beta toxicity [377]. Protection was also provided by dCBP against polyglutamine toxicity in a *Drosophila* Huntington’s disease model [378,379]. Acetylation on histone H3K27 by dCBP is antagonized by pro-aging HDAC Rpd3 [380]. Canonical aversive long-term memory formation in *Drosophila* requires dCBP and this pathway is inhibited by starvation and activated by refeeding [381].

## 15. Nematode Changes in Histone Lysine Acetylation Marks with Aging and Anti-Aging Dietary Restriction

In *C. elegans* MYS-1, a MYST family homolog of yeast Esa1 and mammalian TIP60/KATP5, can increase longevity by acetylating histone H4K16 in the promoter to stimulate expression of the pro-longevity transcriptional regulator *daf-16*, homologous to mammalian FOXO genes [301]. Consistent with this, *mys-1* RNAi decreased lifespan extension in long-lived *daf-2* insulin receptor mutants by decreasing *daf-16* expression [301]. Therefore, activation of MYS-1 leading to increased *daf-16* expression is one mechanism through which increased acetyl-CoA levels may mediate lifespan extension. In mammalian cells, serum deprivation caused the phosphorylation and activation of TIP60/KAT5, which acetylated and activated ULK1/ATG1 increasing the rate of autophagy [382], a second potential anti-aging function. The role of TIP60/KAT5 in aging and neurodegeneration has been reviewed [314].

Acetylation of histone H4K5 in nematodes declined with aging and is regulated by the *C. elegans* homolog of CBP/p300 called CBP-1. Anti-aging dietary restriction (DR) was shown to increase histone H4K5 acetylation levels in worms by increasing the level of CBP-1 [127]. Another group showed that CBP-1 functions in GABAergic neurons to mediate the longevity effects of axenic DR [383]. Whole worm CBP-1 levels did not change with aging [127]. *C. elegans* has three class I HDACs, HDA-1, HDA-2, and HDA-3 that are homologous to Rpd3 in yeast. While *hda-2* and *hda-3* mutant strains are short-lived, knockdown of *hda-2* or *hda-3* by RNA interference resulted in lifespan extension [384]. This suggests that partial, but not full inhibition of class I HDAC activity promotes longevity. Proteomics analysis has shown that HDA-3 levels increased during young and middle adulthood and then fell during late adulthood [385]. Therefore, increased HDA-3 level is one potential mechanism for the decreased histone H4K5 acetylation level that occurs with aging. An aging-induced decrease in nucleocytoplasmic acetyl-CoA levels could also contribute to the decreased histone H4K5 acetylation that occurs in aged worms. Consistent with this hypothesis, mitochondrial ETC dysfunction during larval development has been shown to decrease acetyl-CoA levels in *C. elegans* [386] and ETC dysfunction is a major phenotype of *C. elegans* aging [387]. Proteomics data has shown that the level of acetyl-CoA synthetase ACS-19, homologous to human ACSS2, decreases in post-reproductive worms and this decrease in ACS-19 abundance was delayed in long-lived *daf-2* mutant worms [385].

In *C. elegans* it has yet to be firmly established the complete set of histone lysine residues that are changed with aging or that show altered acetylation to mediate lifespan extension induced by DR or HDAC inhibition. However, addition of the HDAC inhibitors butyrate or trichostatin A increased histone H4K5 acetylation and lifespan in *C. elegans* [127]. Since the mammalian CBP/p300 family is known to strongly acetylate histone H3K18, H3K27 [388], and H3K56 [389], it will be important to determine the effects of HDAC inhibitors and DR on the acetylation of these sites, especially on histone H3K18 in GABAergic neurons where CBP-1 functions to extend lifespan in response to DR [383]. Hyperacetylation of histone H3K18 is associated with inhibition of autophagy in yeast [332,390], so increased histone H3K18 acetylation could possibly block maladaptive neuronal autophagy, a process which contributes to *C. elegans* aging [391,392]. CBP-1 has also been shown to acetylate the pro-longevity DAF-16 transcriptional regulator to exclude it from the nucleus [393]. This acetylation event was inhibited by a catalytically inactive SIR-2.4 deacetylase, a homolog of mammalian SIRT6/7. So, CBP-1 has both pro-longevity and pro-aging roles.

Consistent with CBP-1 possessing a pro-aging role, addition of the CBP-1/p300 inhibitor nordihydroguaiaretic acid (NDGA) decreased histone H3 acetylation, increased autophagy, and extended lifespan in *C. elegans* [394]. Histone H4 acetylation was not monitored. NDGA has also been shown to extend lifespan in male mice [395,396] and fruit flies [397] and was shown to bind and inhibit p300, but not other HATs including GCN5, PCAF, or TIP60/KAT5. In mammalian cells NDGA specifically inhibited histone H3K27 acetylation, but not histone H3K9 acetylation consistent with inhibition of p300, but not GCN5 or PCAF. In male mice NDGA inhibited hypothalamic inflammation and reactive gliosis as a potential mechanism of lifespan extension [116]. As knocking down *C. elegans cbp-1* during adulthood decreases lifespan [127], further lifespan studies should be performed diluting *cbp-1* RNAi clones to diminish knockdown efficacy or using tissue-specific *C. elegans* knockdown strains to attempt to mimic the longevity effect of NDGA administration. It is possible that NDGA increases lifespan by partially inhibiting intestinal CBP-1 activity to enhance autophagy and DAF-16 nuclear localization [393].

The sole GCN5/PCAF family member in *C. elegans* is PCAF-1. At the time of publication studies have yet to be performed to determine a role for PCAF-1 in aging, which is somewhat surprising given that Gcn5 hemizygosity or Gcn5 inhibitors stimulate longevity in yeast [320] and that decreased histone H3K9 acetylation occurs with aging in SAMP8 mouse brains [9]. The HATs present in mammals and the three model organisms discussed above are listed in Table 1 and their known effects on aging are summarized, while the class I and class III HDACs present in these model organisms are listed in Table 2 where their effects on aging are summarized.

## 16. Experiments Using *C. elegans* Suggest That Increased Acetyl-CoA Synthesis Stimulates Longevity

The two major sources of mitochondrial acetyl-CoA are from fatty acid oxidation and glucose-derived pyruvate oxidation. The mitochondrial PDC, composed of E1, E2, and E3 subunits, is inhibited by phosphorylation on three serine residues of the E1 subunit by pyruvate dehydrogenase kinases PDK1-PDK4 in mammals and PDHK-2 in *C. elegans*. Knockdown of *C. elegans pdhk-2* extended lifespan by 20% [414], possibly by increasing the synthesis of acetyl-CoA. Similarly, the PDK inhibitor (PDC activator) dichloroacetate slightly extended lifespan in *C. elegans* [415]. PDC is dephosphorylated and activated by pyruvate dehydrogenase phosphatases PDP1 and PDP2 in mammals and PDP-1 in *C. elegans.* Deficiency of *pdp-1* leading to reduced PDC activity decreased DAF-16 nuclear translocation and lifespan extension in *daf-2* mutants. Overexpression of *pdp-1*, which increases PDC activity, increased lifespan by 17% [416] lending further support to the positive association between PDC activity, acetyl-CoA synthesis, and longevity. This latter data also suggest that increased acetyl-CoA synthesis may stimulate DAF-16 nuclear translocation to mediate lifespan extension. However, no DAF-16 acetylation event has yet been found to stimulate nuclear translocation.

Supplementation of acetate [417], ethanol [418], pyruvate [414], BHB [384], or fatty acids [419] at moderate doses have all been shown to extend lifespan in *C. elegans*. As an example, supplementation of *C. elegans* with the fatty acids stearic acid (50 μg/mL) or linoleic acid (200 μg/mL) increased lifespan up to 32% and 17%, respectively [419]. Acetyl-CoA could be a common downstream metabolite of these compounds that leads to increased histone acetylation to mediate the longevity effects. This hypothesis could be readily tested by determining if knockdown of *acly-1* or *acs-19*/ACSS2 largely prevents metabolite-mediated lifespan extension. Although glucose supplementation to young adult worms decreases lifespan in part due to mitochondrial dysfunction [420], glucose supplementation only during larval development [421] or only during the post-reproductive period [422] extends lifespan, which could be mediated by increased nucleocytoplasmic acetyl-CoA levels and histone acetylation.

TCA cycle metabolite supplementation also increased lifespan in *C. elegans* [423,424]. The only TCA cycle metabolites that were not found to extend lifespan when supplemented were succinyl-CoA, which was not tested, and citrate that showed no effect on lifespan [423]. The lack of pro-longevity effects following citrate supplementation [417] were surprising given that isocitrate extended lifespan. However, citrate may have failed to extend lifespan due to its ability to feedback inhibit PDC, succinate dehydrogenase, and glycolysis [425]. Metabolite supplementation generally showed a hormetic dose response effect on lifespan, and so decreased lifespan frequently occurred at the higher doses tested [423]. This lack of lifespan extension at higher supplemented metabolite concentrations and the failure of citrate to extend lifespan could be due to high cytoplasmic acetyl-CoA levels inhibiting autophagy in the intestine required for lifespan extension.

Another mechanism through which TCA cycle metabolite supplementation may increase lifespan in *C. elegans* is through metabolite imbalance and feedback inhibition of TCA cycle activity. Consistent with this, when malate and fumarate were supplemented to *C. elegans*, the compounds were found to be at least partially metabolized in the opposite direction of normal TCA cycle flux (malate dismutation to fumarate and fumarate conversion to succinate that is excreted). This reverse TCA cycle flux was required for lifespan extension to occur [424]. Inhibited or reversed TCA cycle flux may result in increased levels of isocitrate, citrate, and alpha-ketoglutarate as isocitrate dehydrogenase and alpha-ketoglutarate dehydrogenase are rate limiting steps in the cycle. Increased levels of mitochondrial citrate could lead to its efflux into the cytoplasm where it could stimulate ACLY activity to synthesize acetyl-CoA, which could diffuse into the nucleus and stimulate the activity of HATs such as MYS-1 that enhance expression of DAF-16/FOXO [301].

As in mammals, pantothenate is converted into CoA-SH by a five-step enzymatic process that utilizes four ATP molecules and a cysteine molecule with the first and rate limiting step being catalyzed by pantothenate kinase (*pnk-1* and *pnk-4* in *C. elegans*). The *pnk-1* gene is a DAF-16 target gene [426] and knockdown of *pnk-1* prevented the lifespan extension of the *daf-2* mutant strain [427]. Royal jelly, which is enriched in pantothenate, extended lifespan in *C. elegans* in a DAF-16/FOXO-dependent manner [428]. Knockdown of the CoA-SH synthesis gene T05G5.5/DCAKD, catalyzing the last step of the pathway, induced the UPR^mt^ [429] and activated the pro-longevity SKN-1/Nrf2 transcriptional regulator leading to resistance to oxidative stress [430]. The *acs-19*/ACSS2 gene is also a DAF-16 target gene as identified by chromatin immunoprecipitation and its expression is upregulated 7.7-fold in long-lived *daf-2* mutants [431]. Knockdown of *acs-19*/ACSS2 decreased fat storage and enhanced dauer formation [431]. Knockdown of *acs-19*/ACSS2 caused an initial ROS burst followed by activation of SKN-1/Nrf2 leading to decreased ROS levels [432]. Expression of *C. elegans acs-19*/ACSS2 is specific to the intestine [433], the major metabolic tissue in worms. The data strongly support a pro-longevity role for acetyl-CoA in *C. elegans*.

*C. elegans* has two ATP-citrate lyase genes *acly-1* and *acly-2*. The abundance of the major isoform ACLY-1 did not change substantially with aging, while the abundance of the minor isoform ACLY-2 increased slightly with aging. Both forms showed increased abundance in long-lived *daf-2* mutant worms [385,434], although *acly-2* mRNA levels were decreased in *daf-2* mutants [435]. It still needs to be determined if fatty acid synthesis or histone acetylation plays a greater role in the utilization of this acetyl-CoA for lifespan extension. Activation of the glyoxylate shunt is required for many modalities of lifespan extension in *C. elegans* and the bifunctional ICL-1 protein that constitutes the shunt consumes isocitrate and acetyl-CoA for the synthesis of succinate, malate, and CoA-SH [436]. However, ICL-1 appears to be present in the mitochondrial matrix [437], and therefore it would not consume nucleocytoplasmic acetyl-CoA.

Autophagy in the intestine has been shown to be needed for many paradigms of lifespan extension in *C. elegans* [438]. However, autophagy in adult *C. elegans* neurons has been shown to be pro-aging [392]. In both nematodes and mammalian cells autophagy has been shown to promote longevity under conditions that favor closure of the mitochondrial permeability transition pore (mPTP), but promote aging under conditions that stimulate mPTP opening [439]. The mPTP opens under conditions such as increased matrix space Ca^2+^ levels, increased ROS levels, and increased levels of VDAC1, a peripheral component of the mPTP. Opening of the mPTP results in the induction of autophagy to remove damaged organelles and can also induce cell death if the mPTP opens in sufficient organelles to release a critical amount of cell death-promoting mitochondrial factors to the cytoplasm. *C. elegans acly-1* is abundantly expressed in *C. elegans* neurons [440]. Therefore, part of the lifespan extension mediated by DAF-16 may be mediated by inducing a transcriptional program that both keeps the mPTP closed in neurons by increasing the level of reduced glutathione and other antioxidants and by increasing neuronal cytoplasmic acetyl-CoA levels that inhibit toxic maladaptive autophagy.

Increased AMPK activity has been shown to drive longevity in *C. elegans* [441,442]. The AMPK alpha subunit AAK-2 is required for the lifespan extension mediated by many pro-longevity therapies in nematodes [384] and AAK-2 functions in neurons to drive longevity for some of these therapies [442], but constitutively activated AAK-2 must be expressed in multiple tissues for lifespan extension to occur [441]. It is important to determine if ACLY-1, ACS-19/ACSS2, or altered histone acetylation play roles in different *C. elegans* longevity models including lifespan extension mediated by AMPK activation.

## 17. Decreased Acetyl-CoA during *C. elegans* Development Is Required for the UPR^mt^ to Increase Lifespan

Mitochondrial dysfunction that occurs during larval development activates the ATFS-1 and DVE-1 transcriptional regulators that orchestrate the UPR^mt^ resulting in increased stress resistance and lifespan extension [443,444]. Mitochondrial dysfunction during larval development was shown to result in decreased nucleocytoplasmic acetyl-CoA levels that decreased histone acetylation. The decreased level of acetyl-CoA was required for the proper activation of the UPR^mt^ transcriptional response [386]. Adding back metabolites during the larval development that restored acetyl-CoA levels prevented induction of the UPR^mt^ and lifespan extension. The UPR^mt^ can be initiated by either intestinal or neuronal mitochondrial dysfunction [445]. *C. elegans* HDAC HDA-1 was also shown to be required for the induction of UPR^mt^ [446], which further supports a role for decreased histone acetylation in this process. So, low levels of nucleocytoplasmic acetyl-CoA during *C. elegans* development drive longevity through UPR^mt^ activation, while increased levels or activity of acetyl-CoA synthesis enzymes during adulthood correlates with increased longevity. Complexly, CBP-1 was also shown to be essential for UPR^mt^-mediated longevity [126] as well as for the extended longevity caused by increased temperature during larval growth [447]. This temporary slight temperature stress (of only 25 °C) induced a long-lasting increase in histone H4 acetylation that was associated with lifespan extension. Therefore, even though stressors during larval development differentially affect histone acetylation, CBP-1 appears to be a conserved downstream mediator of the longevity response. It is possible that CBP-1 is able to function at lower acetyl-CoA levels than other HATs. In this way CBP-1 can acetylate histones to stimulate UPR^mt^-induced longevity at a time when nucleocytoplasmic acetyl-CoA levels and global histone acetylation levels decline. However, the levels of acetyl-CoA could increase in only a small subset of *C. elegans* cells, such as in GABAergic neurons, to activate CBP-1 to promote UPR^mt^-induced longevity, while acetyl-CoA levels decline in other tissues. 

## 18. Conclusions

Although nucleocytoplasmic acetyl-CoA metabolism and histone acetylation have been studied for many years, their detailed roles in the aging process remain unclear. Cytoplasmic acetyl-CoA clearly plays a pro-aging role in many tissues through inhibition of autophagy. CR and high-fat, low carbohydrate diets, such as the ketogenic diet, decrease the expression of SREBP-1 to decrease ACLY and ACSS2 expression and acetyl-CoA levels to increase autophagy and promote longevity. But, hypothalamic nuclear CBP and PCAF likely utilize acetyl-CoA in pro-longevity roles and the ability of ACSS2 to associate with HATs that acetylate histone H3K9 and H3K27 in chromatin likely facilitates these activities. Increased AMPK activity in glia stimulates ACSS2 nuclear translocation to increase expression of TFEB target genes that stimulate autophagy. But under standard conditions most neuronal ACSS2 is already localized to the nucleus. Since PDK2 and NF-κB play pro-inflammatory and pro-aging roles in glial cells, the AMPK-ACSS2-TFEB pathway likely stimulates autophagy in hypothalamic astrocytes to decrease inflammation and slow the rate of aging [448]. Further research is needed (1) to elucidate the mechanisms through which ACSS2 mediates these opposing effects on autophagy in different tissues, (2) to decipher the mechanisms through which HDAC inhibitors delay aging, (3) to determine the mechanisms through which CR increases CBP expression in the hypothalamus, (4) to determine if hypothalamic CBP-mediated histone acetylation promotes longevity in mammals by inducing the UPR^mt^, and (5) to determine if GTA or other acetate-releasing compounds that increase brain histone acetylation can mimic the anti-aging effects of HDAC inhibitors and CR. If acetate-releasing compounds are administered during fasting, they may selectively increase nuclear acetyl-CoA levels and histone acetylation in the hypothalamus and other brain regions to induce an anorexigenic and anti-aging gene expression program without increasing cytoplasmic acetyl-CoA levels in peripheral tissues to inhibit autophagy. Through the use of blood-brain barrier-permeable AMPK activators, acetate-releasing compounds, and HDAC inhibitors it may be possible to induce relatively long-lasting increases in hypothalamic histone acetylation to increase the expression of genes that increase autophagy, decrease inflammation, and slow organismal aging.

## Figures and Tables

**Table 1:** HATs and their involvement in aging in model organisms.

HAT Protein	Organism	Involvement in Aging
p300/CBP superfamily		
-	*S. cerevisiae*	-
CBP-1	*C. elegans*	DR requires CBP-1 for lifespan extension and increases acetylation of histone H4K5 [127]. NDGA inhibits CBP-1 to increase lifespan and decrease histone H3 acetylation [394].
dCBP/nejire	*D. melanogaster*	
p300, CBP	mammals	Hypothalamic CBP expression correlates with lifespan in studies with five mouse strains [127].
GNAT superfamily		
Gcn5, Hat1, Nut1, Elp3, Hpa2, Hpa3, Nat4	*S. cerevisiae*	Deletion of Gcn5 decreases CLS [315,316,317]. Gcn5 hemizygosity increases RLS [320]. Nat4 deletion increases RLS [340].
PCAF-1, HAT-1, ELPC-3	*C. elegans*	
dGcn5, Elp3, Atac2	*D. melanogaster*	
GCN5, PCAF, HAT1, ELP3, ATF2, ATAC2	mammals	HAT1 hemizygosity causes premature aging [151].
MYST superfamily		
Esa1, Sas2, Sas3	*S. cerevisiae*	Deletion of Sas2 increases RLS by decreasing histone H4K16 acetylation [311]. Esa1 acetylates histone H4K5 and H4K12 to oppose pro-aging HDAC Rpd3 [398].
MYS-1, MYS-2, LSY-12, MYS-4	*C. elegans*	MYS-1 is required for the expression of *daf-16* [301].
Mof, Myst5/CG1894, Tip60, Enok, Chameau	*D. melanogaster*	Chameau hemizygosity increases lifespan and decreases histone H4K12 acetylation [375].
KAT5, KAT6A, KAT6B, KAT7, KAT8	mammals	Inhibitors of KAT6A/B induce senescence [399], while knockout of KAT7 prevents senescence [400].
Other		
Rtt109	*S. cerevisiae*	Deletion of Rtt109 decreases RLS (but not CLS [401]) and histone H3K56 acetylation [311].

**Table 2:** Class I and III HDACs and their involvement in aging in model organisms.

HDAC Protein	Organism	Involvement in Aging
Class I		
Rpd3, Hos1, Hos2, Hos3	*S. cerevisiae*	Rpd3 deletion increases RLS [342].
HDA-1, HDA-2, HDA-3	*C. elegans*	Knockdown of *hda-2* or *hda-3* extends lifespan, while knockout decreases lifespan [384].
Rpd3	*D. melanogaster*	Rpd3 hemizygosity increases lifespan [362].
HDAC1, HDAC2, HDAC3, HDAC8	mammals	HDAC inhibitors are protective in many models of aging-related disease [402,403].
Class III (nucleocyto)		
Sir2, Hst1, Hst2, Hst3, Hst4	*S. cerevisiae*	Sir2 deletion decreases RLS [311]. Hst3 deletion decreases RLS [404] or CLS [405]. Hst1 deletion decreases CLS [405].
SIR-2.1, SIR-2.4	*C. elegans*	SIR-2.1 promotes lifespan extension [406]. SIR-2.4 induces DAF-16 nuclear localization [393].
dSir2, dSirt2, dSirt6, dSirt7	*D. melanogaster*	dSir2 was shown to promote longevity [407,408]. Neuronal dSirt6 knockdown shortens lifespan [409].
SIRT1, SIRT2, SIRT6, SIRT7	mammals	Overexpression of SIRT1 in brain extends lifespan [98]. Overexpression of SIRT6 extends lifespan [290], while knockout decreases lifespan [291].
Class III (mito)		
Hst4	*S. cerevisiae*	Hst4 deletion does not affect RLS [404] or CLS [405].
SIR-2.2, SIR-2.3	*C. elegans*	Mutants of *sir-2.2* and *sir-2.3* are long-lived under certain dietary conditions [410].
dSirt2 [411], dSirt4	*D. melanogaster*	Knockout of dSirt4 decreases lifespan, while overexpression increases lifespan [412]. Knock-down of dSirt2 in neurons decreases lifespan [409].
SIRT3, SIRT4, SIRT5	mammals	SIRT3 is required for some of the cytoprotection provided by CR [413].
